# TLR4-mediated pyroptosis in human hepatoma-derived HuH-7 cells induced by a branched-chain polyunsaturated fatty acid, geranylgeranoic acid

**DOI:** 10.1042/BSR20194118

**Published:** 2020-04-28

**Authors:** Suemi Yabuta, Yoshihiro Shidoji

**Affiliations:** Molecular and Cellular Biology, Graduate School of Human Health Science, University of Nagasaki, 1-1-1 Academy Hills, Nagayo, Nagasaki 851-2195, Japan

**Keywords:** caspase-4, gasdermin D, lipid-induced UPR, mitochondrial ROS, nuclear factor kappaBactor kappaB, pyroptosis

## Abstract

A branched-chain polyunsaturated fatty acid, geranylgeranoic acid (GGA; C_20:4_), which is an endogenous metabolite derived from the mevalonate pathway in mammals, has been reported to induce cell death in human hepatoma cells. We have previously shown that the lipid-induced unfolded protein response (UPR) is an upstream cellular process for an incomplete autophagic response that might be involved in GGA-induced cell death. Here, we found that Toll-like receptor 4 (TLR4)-mediated pyroptosis in HuH-7 cells occurred by GGA treatment. The TLR4-specific inhibitor VIPER prevented both GGA-induced cell death and UPR. Knockdown of the *TLR4* gene attenuated GGA-induced cell death significantly. Upon GGA-induced UPR, caspase (CASP) 4 (CASP4) was activated immediately and gasdermin D (GSDMD) was translocated concomitantly to the plasma membrane after production of the N-terminal fragment of GSDMD. Then, cellular CASP1 activation occurred following a second gradual up-regulation of the intracellular Ca^2+^ concentration, suggesting that GGA activated the inflammasome. Indeed, the mRNA levels of NOD-like receptor family pyrin domain containing 3 (*NLRP3*) and interleukin-1 β (*IL1B*) genes were up-regulated dramatically with translocation of cytoplasmic nuclear factor-κB (NF-κB) to nuclei after GGA treatment, indicating that GGA induced priming of the NLRP3 inflammasome through NF-κB activation. GGA-induced up-regulation of CASP1 activity was blocked by either oleic acid, VIPER, MCC950 (a selective inhibitor of the NLRP3 inflammasome), or CASP4-specific inhibitor peptide cotreatment. Pyroptotic cell death was also confirmed morphologically by bleb formation in time-series live cell imaging of GGA-treated cells. Taken together, the present results strongly indicate that GGA causes pyroptotic cell death in human hepatoma-derived HuH-7 via TLR4 signalling.

## Introduction

A 20-carbon branched-chain polyunsaturated fatty acid or acyclic diterpenoid acid of geranylgeranoic acid (GGA) was first recognised as an acyclic retinoid because the acid showed agonist activity as a ligand for retinoic acid receptor-β, retinoid-X receptor-α, and cellular retinoic acid-binding protein [1]. However, GGA has an anti-tumour action that is different from that of natural retinoids. Micromolar concentrations of GGA induce cell death in human hepatoma cells, but not in mouse primary hepatocytes [2], although natural retinoids, such as all-*trans* and 9-*cis* retinoic acid, do not induce cell death in hepatoma cells, indicating that a non-retinoidal function of GGA may be important for cancer prevention [3]. Thereafter, we identified natural GGA in medicinal herbs [4], suggesting that GGA might be better classified as a biologically active diterpenoid rather than a retinoid. Recently, we reported that GGA is biosynthesised via the mevalonate pathway in mammalian cells including human cells by isotopomer spectral analysis using ^13^C-labelled mevalonolactone [5].

GGA-induced tumour-specific cell death was first characterised as apoptosis, which was evidenced by chromatin condensation and nucleosomal ladder formation [3]. However, N-acetyl-aspartyl-glutamyl-valyl-aspartyl-aldehyde (Ac-DEVD-CHO), a specific inhibitor of caspase (CASP)-3/7, was unable to block GGA-induced cell death, indicating that GGA did not induce typical apoptosis, but rather caspase-3/7-independent cell death [2]. Next, we investigated another form of programmed cell death, autophagic cell death, after GGA treatment. As a result, GGA at micromolar concentrations induced an incomplete autophagic response characterised by massive accumulation of initial/early autophagosomes and defective autolysosome formation or impaired fusion of autophagosomes with lysosomes [6]. Furthermore, GGA-induced cell death was accompanied by increased production of reactive oxygen species (ROS) such as superoxides in mitochondria [6] and delayed dissipation of the mitochondrial inner membrane potential (ΔΨ*m*) [2]. Interestingly, wortmannin, a broadly specific inhibitor of phosphoinositide 3-kinases, prevented the GGA-induced incomplete autophagic response as well as hyperproduction of superoxides in mitochondria [6], suggesting that superoxide hyperproduction might be mediated through signal transduction rather than the direct action of GGA in mitochondria. Notably, α-tocopherol, a fat-soluble antioxidant vitamin, efficiently prevented ΔΨ*m* dissipation and GGA-induced cell death [2]. This suggested that mitochondrial superoxide hyperproduction might be indispensable for GGA-induced cell death.

Next, we focused on which cellular events were induced initially by GGA as an upstream signal for the incomplete autophagic response. We found that GGA immediately provoked a lipid-induced endoplasmic reticulum (ER) stress response/unfolded protein response (UPR) that was linked to its lipotoxicity in human hepatoma cells [7]. As a general characteristic of lipid-induced UPR, GGA-induced UPR and cell death were also suppressed by cotreatment with equimolar oleic acid [7]. Currently, at least two hypotheses have been reported to describe the mechanism of oleate-mediated suppression of lipid-induced UPR. First, phospholipids containing monounsaturated oleic acids inserted in the ER membrane inhibit lipid (e.g., palmitic acid)-induced UPR by increasing membrane fluidity [8,9]. Second, oleic acid promotes lipid droplet formation, thereby sequestrating UPR-causing lipids such as palmitic acid from the ER membrane to lipid droplets [10,11]. In either case, oleic acid must first be thioesterified by coenzyme A (CoA)-SH to become oleyl-CoA, the only substrate of the enzymatic reaction into which oleic acid is introduced to either phospholipids in the ER or triacylglycerols in lipid droplets. However, although the carboxyl group of oleic acid is blocked by a methyl group, the inhibitory effect of the resultant methyl oleate on GGA-induced UPR is similar to that of oleate [7]. Furthermore, the preventive effect of oleic acid on GGA-induced UPR was not observed when it was added before GGA treatment [7]. Therefore, we speculated that oleic acid might directly or competitively block GGA-mediated signals to induce UPR and cell death.

Thus, the next issue was how GGA induced UPR in hepatoma cells. A previous study described the Toll-like receptor-4 (TLR4)/UPR axis [12], in which palmitate-enriched high fat diet-mediated stimulation of TLR4 signalling caused UPR in mice. Since then, several studies have reported that saturated fatty acid-mediated TLR4 signalling is an upstream signal that induces ER stress, UPR, and mitochondrial hyperproduction of superoxides [13–15]. This indicates the existence of a novel signalling network that links TLR4 activation, ER stress, and mitochondrial dysfunction [12,13]. Another line of evidence for the TLR4/UPR axis is that 7-ketocholesterol-induced inflammation is mediated mostly through the TLR4 receptor and involves a robust UPR that appears to be mediated by as yet unidentified kinases activated through the TLR4 receptor [16]. Both saturated fatty acids and oxidised cholesterols as lipids induce UPR [17,18]. However, the molecular mechanism of lipid-induced UPR is still controversial. Therefore, it would be interesting to determine whether another novel UPR-inducing lipid such as GGA stimulates TLR4 signalling to induce UPR.

Finally, how GGA induces cell death in hepatoma cells is unclear. Our previous study reported that CASP1 inhibitor N-acetyl-tyrosyl-valyl-alanyl-aspartyl-chloromethylketone (Ac-YVAD-CMK) blocked GGA-induced cell death [2], indicating activation of inflammasomes upon GGA treatment because CASP1 activation is the main output of the inflammasome [19]. Prior to the activation, inflammasome priming consisting of transcriptional up-regulation of NOD-like receptor family pyrin domain containing 3 (*NLRP3*) and interleukin 1 β (*IL1B*) genes is required [20]. TLR4 signalling is involved in priming of inflammasomes [20,21] and mitochondrial ROS production [22]. Furthermore, inflammasomes drive pyroptosis, a lytic form of cell death [23] involving a mechanism partly mediated by gasdermin D (GSDMD), a CASP1-cleavable target [24,25]. In addition to CASP1, GSDMD can be cleaved at Asp^276^ by caspase-4/5 (CASP4/5, human orthologues of rodent caspase-11) [26]. The N-terminal fragment of GSDMD was recently reported to be the sole executor of pyroptotic cell death with an intrinsic pore-forming activity in the plasma membrane [27,28].

Based on these studies, we hypothesised that GGA stimulates TLR4 signalling to induce UPR, activates CASP1, and then induces pyroptotic cell death in human hepatoma cells. In the present study, we used the TLR4-specific inhibitor VIPER [29] and siRNA against the *TLR4* gene to demonstrate that GGA-induced UPR and cell death are both driven by TLR4 signalling. Furthermore, we show that GGA-induced hyperproduction of mitochondrial superoxide is dependent on TLR4 signalling and indispensable for GGA-induced pyroptotic cell death using antioxidant vitamin α-tocopherol.

## Materials and methods

### Chemicals

GGA was a generous gift from Kuraray (Okayama, Japan). α-Tocopherol, oleic acid, thapsigargin, tunicamycin, C34 (TLR4 inhibitor), *TLR4* siRNA (duplex of Hs TLR4 2250 s and as), and scrambled RNA (duplex of Mission SIC-001 s and as) were purchased from Sigma–Aldrich (St. Louis, MO, U.S.A.). TLR4 inhibitors TAK242 and VIPER (control peptide: CP7) were obtained from ChemScene Chemicals (Monmouth Junction, NJ, U.S.A.) and Novus Biologicals (Littleton, CO, U.S.A.), respectively. NLRP3 and nuclear factor-κB (NF-κB) activation inhibitors MCC950 (CP-456773), BAY11-7082 (#T2846), and BI605906 (HY-1309) were from Selleck Chemicals (Houston, TN, U.S.A.), Tokyo Chemical Industry (Tokyo, Japan), and MedChemExpress (Monmouth Junction, NJ 08852, U.S.A.), respectively. CASP4 inhibitor Z-LEVD-FMK was obtained from BioVision (Milpitas, CA, U.S.A.). Merck™ RIPA Lysis Buffer, 10× was purchased from Thermo Fisher Scientific.

### Cell culture

Human hepatoma-derived HuH-7 cells were obtained from the RIKEN BioResource Center (Tsukuba, Japan) and cultured in high glucose Dulbecco’s modified Eagle’s medium (DMEM; Wako Pure Chemical Industries) supplemented with 5% foetal bovine serum (FBS; HyClone Laboratories, Thermo Fisher Scientific, Waltham, MA, U.S.A.). HuH-7 cells were cultured in DMEM containing 5% FBS for 2 days followed by serum-free DMEM for a further 2 days before drug treatments. GGA in ethanol was dispersed in serum-free medium.

### RT-qPCR

Total RNA was isolated using a High Pure RNA Isolation Kit (Roche Diagnostics, Basel, Switzerland). cDNA was generated using a Transcriptor First Strand cDNA Synthesis kit with random hexamers (Roche). Nucleotide sequences of the PCR primers for *XBP1s, DDIT3, NLRP3, IL1B, CASP4/5, TLR1/2/4/6/9*, and *28S rRNA* cDNAs are listed in Supplementary Table S1. Real-time PCR was performed with Roche Faststart DNA Master SYBR Green I (Roche) and cDNA on a LightCycler 1.5 or 96 (Roche).

### Western blotting

Equal amounts (40 µg) of proteins prepared by cell lysis in RIPA buffer were separated by SDS/PAGE. The blotted membranes were probed with a rabbit polyclonal antibody against CASP4 (Cell Signaling Technology, Boston, MA, U.S.A.) or GSDMD (Abcam, Cambridge, U.K.). A horseradish peroxidase (HRP)-labelled secondary antibody (GE Healthcare, Tokyo, Japan) was detected with Western Chemiluminescent HRP substrate (Merck Millipore, Japan) or SuperSignal West Femto maximum sensitivity substrate (Thermo Fisher Scientific) using an ImageQuant LAS4000 (GE Healthcare, Tokyo, Japan).

### Immunofluorescence microscopy

Immunofluorescence staining was performed using cultured HuH-7 cells grown on glass inserts in a 24-well plate. Briefly, after fixation, the cells were incubated at 4°C overnight with anti-GSDMD (Cell Signaling Technology) or anti-nuclear factor-κB (NF-κB) p65 (D14E12, Cell Signaling Technology) antibodies, followed by 1 h of incubation with Alexa 488-labelled goat anti-rabbit IgG (Invitrogen, Molecular Probes, Tokyo, Japan) or Alexa Fluor 568–conjugated goat anti-mouse IgG (Invitrogen). After rinsing with PBS (−), the cells were mounted in PermaFluor (Beckman Coulter, La Brea, CA, U.S.A.) containing DAPI on a glass slide and observed under a confocal laser-scanning fluorescence microscope (LSM700 2Ch URGB with Axio Observer Z1 Bio; Carl Zeiss, Göttingen, Germany).

### Live cell imaging

HuH-7 cells were cultured in glass-bottomed dishes (Matsunami Glass, Osaka, Japan) in a chamber unit (INUG2-ZIL; Tokai Hit, Hamamatsu, Shizuoka, Japan) equipped to the LSM700 inverted laser-scanning confocal fluorescence microscope. Time-series data of fluorescence and DIC images was obtained for Ca^2+^ and mitochondrial superoxide measurements using a fluorogenic dye, Fluo-4 AM (Dojindo, Kumamoto, Japan), or MitoSOX™ Red (Thermo Fisher Scientific), respectively. Fluo-4 experiments were performed after GGA treatment in serum-free DMEM or serum/Ca^2+^-free DMEM. The mean pixel intensity of Fluo-4 fluorescence for each cell was determined in regions of interest (ROIs) of the time-lapse image series using Zen 2010 B SP1 software (Carl Zeiss). ROIs were used to measure the raw fluorescence that was then converted into relative intensity (each raw fluorescence divided by 0-h fluorescence).

### Cell viability and cytotoxicity assays

Cells were seeded into a 96-well plate. A CellTiter-Glo assay kit (Promega KK, Tokyo, Japan) was used to determine the number of viable cells by measuring the cellular ATP levels, according to the manufacturer’s instructions. Luminescence was recorded using a Centro XS^3^ LB960 (Berthold Technologies, Wildbad, Germany). Cytotoxicity was assayed by determining lactate dehydrogenase (LDH) activity in the culture medium with a Cytotoxicity Detection kit (LDH) (Roche Diagnostics).

### CASP1 activity assay

A Caspase-Glo1 assay kit (Promega) was used to measure the activity of CASP1 in cells seeded in a 96-well plate. Luminescence was recorded using the Centro XS^3^ LB960.

### Statistical analysis

All values are expressed as the mean ± SD. Significant differences among each series of experiments were examined by the Student’s *t* test. *P*<0.05 was considered as statistically significant.

## Results

### GGA-induced cell death is mediated by TLR4 signalling

**Figure 1:**
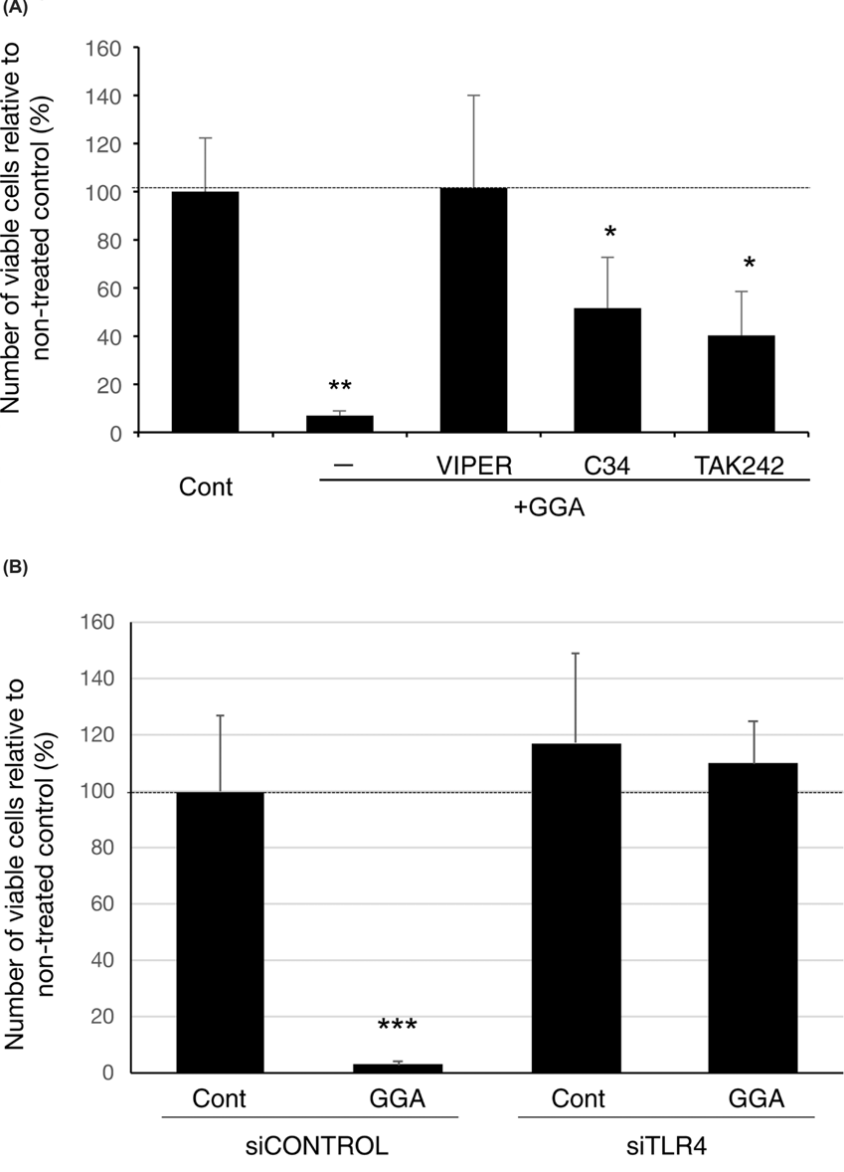
TLR4 is involved in GGA-induced cell death of HuH-7 cells (**A**) HuH-7 cells were treated with 20 μM GGA in the presence of 5 μM TLR4-specific inhibitor, VIPER, C34, or TAK242 for 24 h. (**B**) HuH-7 cells were treated with 0 (vehicle control) or 20 µM GGA for 24 h, after 72 h of pretreatment with control siRNA (siCONTROL) or 10 μM TLR4 siRNA (siTLR4). Viable cell numbers were determined by measuring cellular ATP contents with a CellTiter-Glo assay kit. The y-axis represents the viable cell number as a % of untreated control cell numbers or relative chemiluminescence intensity. Values are the means ± SD (*n*=3, 6, or 8). ** and *** indicate statistical significance (*P*<0.01 and *P*<0.001, respectively), compared with controls and * indicates statistical significance (*P*<0.05) compared with GGA alone as determined by the Student’s *t* test.

As shown in Figure 1A, GGA alone induced cell death in more than 90% of HuH-7 cells at 24 h after treatment. VIPER peptide prevented GGA-induced cell death dose-dependently in comparison with control heptapeptide CP7 (Supplementary Figure S1). Cotreatment with 5 µM VIPER blocked GGA-induced cell death, but two other TLR4-specific inhibitors, C34 and TAK-242, only partially but significantly prevented GGA-induced cell death. To confirm that TLR4 was involved in GGA-induced cell death, TLR4 was knocked down in HuH-7 cells by siRNA. Expression of the *TLR4* gene was knocked down in a dose-dependent manner by *TLR4* siRNA (siTLR4) (Supplementary Figure S2). GGA-induced cell death was attenuated significantly at 72 h after treatment with 10 nM siTLR4, but not the siControl (scrambled siRNA) (Figure 1B).

### GGA-induced UPR is mediated by TLR4 signalling

**Figure 2:**
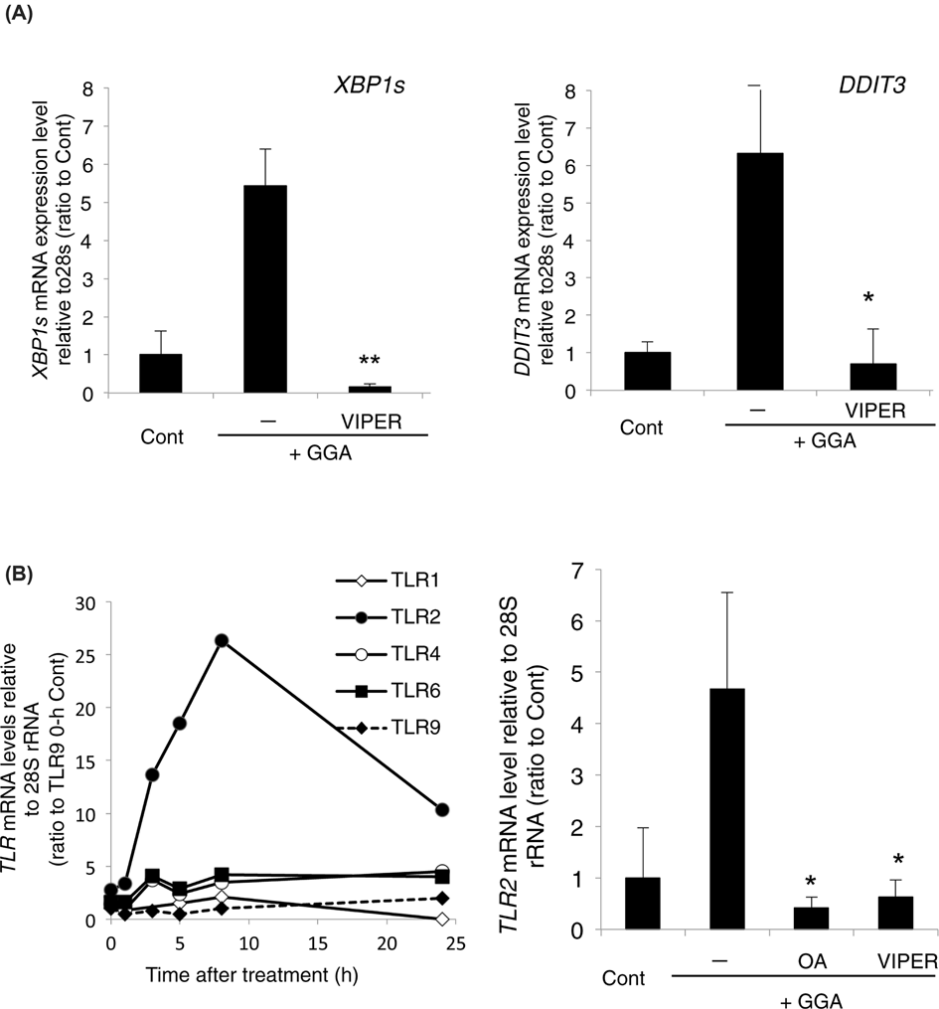
GGA-mediated TLR4 signalling induces UPRs (**A**) Levels of *XBP1s* (left panel) and *DDIT3* mRNA (right panel) were measured by RT-qPCR at 5 h after 20 μM GGA treatment in the absence (−) or presence of 5 μM VIPER. Values are the means ± SD (*n*=3). Control cells were treated with the ethanol vehicle alone. * and ** indicate statistical significance (*P*<0.05 and *P*<0.01, respectively) compared with GGA alone as determined by the Student’s *t* test. (**B**) *Left panel*: total RNA was extracted to measure the levels of *TLR*1, 2, 4, 6, and 9 mRNAs by RT-qPCR at the indicated time points after treatment with 20 µM GGA; *Right panel*: HuH-7 cells were treated with 20 µM GGA for 5 h in the absence (−) or presence of 50 µM oleic acid (OA) or 5 µM VIPER. Control cells were treated with the vehicle alone. Values are the means ± SD (*n*=3). * indicates statistical significance (*P*<0.05) compared with GGA alone as determined by the Student’s *t* test.

We recently reported that GGA induces UPR immediately, which is essential for GGA-induced cell death [7]. Therefore, we next observed the effect of VIPER cotreatment on GGA-induced UPR. VIPER suppressed up-regulation of *XBP1s* and *DDIT3* mRNA expression significantly and completely (Figure 2A), both of which are hallmarks of GGA-induced UPR [7]. Because VIPER is a TLR4-specific inhibitor, some TLR4 signals are thought to be involved in GGA-induced UPR.

Among TLR family members, *TLR2* gene expression is known to be up-regulated via ATF4 [30,31], a downstream component of the PERK branch of UPR. Therefore, we measured expression of the *TLR2* gene and some other TLR family members during GGA-induced UPR. As expected, based on the GGA-induced up-regulation of *DDIT3* gene expression (Figure 2A), a downstream signal of the PERK pathway, GGA up-regulated (>25-fold) *TLR2* gene expression markedly in a time-dependent manner (Figure 2B). Expression of the other two TLR family members, namely *TLR4* and *TLR6* genes, was increased slightly by three- to four-fold after GGA treatment, but the mRNA levels of *TLR1* and *TLR9* were down-regulated after GGA treatment. The baseline expression of each TLR family member is shown in Supplementary Figure S3, relative to that of the *TLR9* gene, where the mRNA level of the *TLR2* gene was the highest and significantly higher than that of the *TLR9* gene.

In addition to the inhibitory effects of VIPER and oleic acid [7] on both GGA-induced cell death and UPR, VIPER and oleic acid suppressed the GGA-induced up-regulation of the *TLR2* gene (Figure 2B, right panel), suggesting that TLR2 might be involved in GGA-induced cell death. However, lipoteichoic acid (5 µM) [32], a TLR2-specific agonist, and *o*-vanillin (5 µM) [33], a TLR2-specific antagonist, did not induce cell death without GGA and did not prevent GGA-induced cell death, respectively (unpublished observations). Therefore, we considered that VIPER simply suppressed GGA-induced up-regulation of *TLR2* gene expression because it suppressed GGA-induced UPR. Indeed, cotreatment with oleic acid, an inhibitor of GGA-induced UPR [7], also prevented GGA-induced up-regulation of *TLR2* gene expression (Figure 2B), implying that GGA-induced UPR is a direct upstream signal for the up-regulation of *TLR2* gene expression.

Although GGA-induced up-regulation of *TLR2* gene expression did not appear to be linked to GGA-induced cell death, GGA-induced UPR itself may be a signal related to cell death directly. However, α-tocopherol, which prevents GGA-induced cell death [2], did not suppress, but even enhanced GGA-induced UPR (Supplementary Figure S4). Therefore, we suggest that TLR4 signalling is involved in both GGA-induced UPR and GGA-induced cell death specifically and that GGA-induced UPR alone does not cause such cell death.

### Up-regulation of the CASP4 active form and immediate translocation of GSDMD after GGA treatment

**Figure 3:**
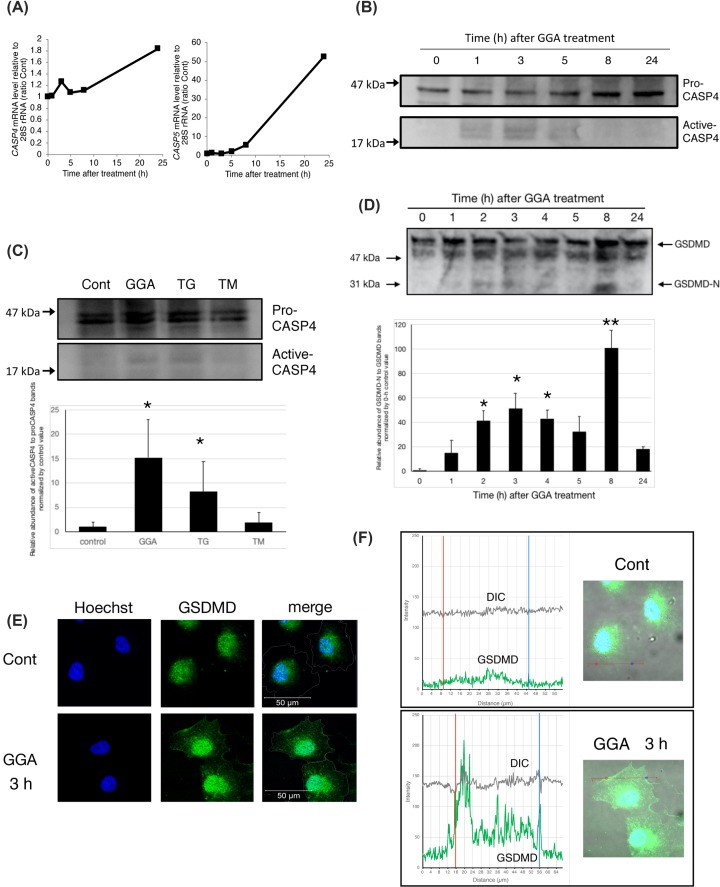
GGA-induced UPR activates caspase-4 and induces plasma membrane translocation of GSDMD in HuH-7 cells (**A**) Total RNA was extracted to measure the levels of *CASP4* or *CASP5* mRNAs by RT-qPCR at the indicated times after treatment with 20 µM GGA (*n*=3). (**B**) Whole cell lysates were prepared at the indicated time points after GGA treatment, and pro-CASP4 (45 kDa) and active CASP4 (20 kDa) levels were detected by Western blotting. The experiment was repeated three times and a representative blot is shown. (**C**) HuH-7 cells were treated for 5 h with either 20 μM GGA, 25 ng/ml thapsigargin (TG), or 0.25 μg/ml tunicamycin (TM). Western blot analysis was performed three times. After quantitative analysis of each band by ImageJ, the average values of relative abundance of active CASP4 to pro-CASP4 bands normalised to the 0-h control value are depicted with standard error bars (*n*=3). *P<0.05 versus 0-h control. (**D**) HuH-7 cells were treated with 20 μM GGA for 0, 1, 2, 3, 4, 5, 8, and 24 h. Then, the N-terminal fragment (30 kDa, GSDMD-N) of GSDMD was analysed by Western blotting with an anti-GSDMD antibody in comparison with intact GSDMD (50 kDa). The experiment was repeated three times. After quantitative analysis of each band by ImageJ, the average values of relative abundance of GSDMD-N to full-length GSDMD bands normalised to the 0-h control value are depicted with standard error bars (*n*=3). *P<0.05, **P<0.001 versus 0-h control. (**E**) Immunofluorescence images were obtained with an anti-GSDMD antibody, and nuclei were counterstained with Hoechst 33258 (blue) at 3 h after GGA treatment. Merged images (merge) were constructed by tracing cell membranes on differential interference contrast (DIC) images. (**F**) Based on the merged images of GSDMD (green), nuclei, (blue), and DIC (grey), the profile view of Zen software provided the intensity curves of green fluorescence (GSDMD) and grey (DIC) on each straight line drawn in the merged images (right panels). The pair of red and blue vertical lines in each graph (left panels) indicate the positions of the same coloured dots on each straight line drawn in the merged image (right panels).

As described above, we found that GGA causes UPR via TLR4 signalling, which may be linked to cell death in hepatoma cells. Next, we investigated ER-resident CASP4 dynamics [34,35]. First, we analysed GGA-induced changes in the mRNA levels of *CASP4* and *CASP5* genes, both of which are human orthologues of rodent caspase-11. The mRNA levels of both genes began to increase time-dependently at 5 h after addition of GGA. Expression of the *CASP4* gene was almost twice the normal level and *CASP5* gene expression was increased by 55-fold at 24 h (Figure 3A). Because the baseline mRNA level of the *CASP4* gene relative to 28S rRNA was ≥200-fold that of the *CASP5* gene, we measured the protein levels of CASP4 after GGA treatment. In accordance with the changes in *CASP4* mRNA levels, the pro-CASP4 protein (45 kDa) level was almost doubled at 24 h after GGA treatment (Figure 3B). More importantly, active CASP4 bands (20 kDa) were detected from 1 to 5 h after the addition of GGA and disappeared after 8 h, indicating that GGA induced rapid activation of CASP4 and subsequently suppressed its activation. ER Ca^2+^-releasing thapsigargin, a representative inducer of ER stress, also significantly induced production of active CASP4, probably through ER stress, whereas N-glycosylation-inhibiting tunicamycin, another authentic inducer of ER stress, did not activate CASP4 (Figure 3C). These results suggested that Ca^2+^ released from ER may be important for CASP4 activation, which is consistent with a previous report [35]. Interestingly, thapsigargin induced rapid UPR and up-regulated the expression of *CASP4, CASP5*, and *TLR2* mRNAs in a time-dependent manner that was slightly different from GGA (Supplementary Figure S5). Furthermore, considering that the induction of UPR by thapsigargin was not inhibited by cotreatment with oleic acid or VIPER (Supplementary Figure S6), Ca^2+^-releasing UPR itself may cause up-regulation of *CASP4/5* and *TLR2* mRNAs without TLR4 signalling.

Because CASP4 was activated, we expected that GSDMD cleavage should occur. Indeed, as shown in Figure 3D, we detected an amino-terminal GSDMD-N fragment (30 kDa) by Western blotting as early as 1 h after GGA addition and the fragment was detected at its maximum at 8 h with a transient peak at 3 h. In accordance with this biochemical change, the subcellular distribution of GSDMD was altered after GGA treatment. Although most GSDMD remained in the nucleus of 3-h ethanol-treated control cells, unexpectedly, the migration of GSDMD from the nucleus to the plasma membrane was observed clearly at 3 h after the addition of GGA (Figure 3E), where GSDMD was distributed intensely in the concave parts of the plasma membrane (red and blue dots and lines in Figure 3F, lower panel). However, to date, we have not detected signs of cell death in human hepatoma cells in culture within 6 h of GGA treatment by Trypan Blue exclusion, the mitochondrial function of succinate dehydrogenase, or cellular ATP content.

### Activation of CASP1 activity after GGA treatment to induce pyroptosis

**Figure 4:**
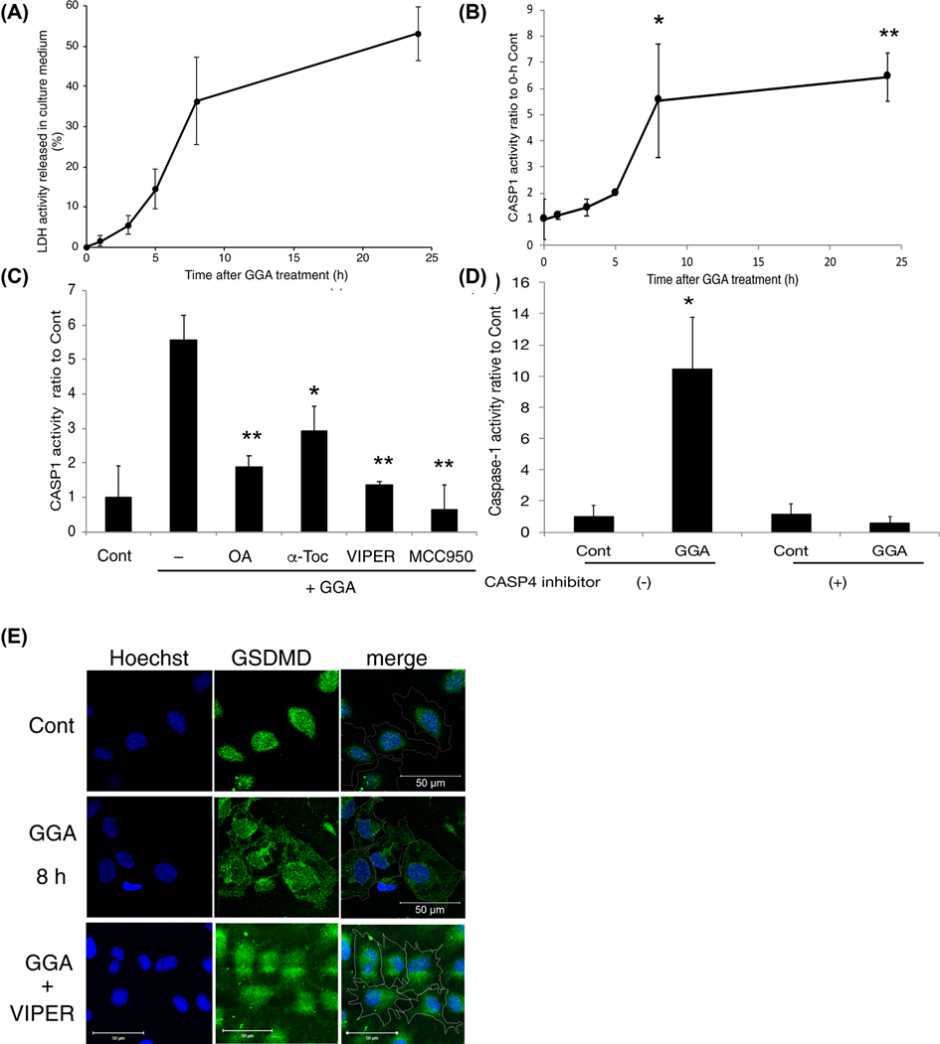
GGA induces activation of CASP1 after CASP4 activation (**A**) LDH activity in the culture medium at the indicated time points after GGA treatment was measured by an LDH Cytotoxicity Detection kit. (**B**) CASP1 activity in cell lysates was estimated by a Caspase-Glo 1 assay kit at the indicated time points after 20 µM GGA treatment. Each point represents the mean ± SD (*n*=3). * and ** indicate statistical significance (*P*<0.05 and *P*<0.01, respectively) compared with 0-h controls as determined by the Student’s *t* test. (**C**) HuH-7 cells were treated with 20 μM GGA in the presence of 50 μM oleic acid (OA), 100 μM α-tocopherol (α-Toc), 5 μM VIPER, or 5 μM MCC950 for 8 h. CASP1 activity was measured in each cell lysate. Each point represents the mean ± SD (*n*=3). * and ** indicate statistical significance (*P*<0.05 and *P*<0.01, respectively) versus GGA alone. (**D**) HuH-7 cells were treated with 20 μM GGA in the presence or absence of 5 μM caspase-4 inhibitor Z-LEVD-FMK for 8 h. After the treatment, CASP1 activity was measured in each cell lysate. Each point represents the mean ± SD (*n*=3). * indicates statistical significance (*P*<0.05) versus each control. (**E**) Immunofluorescence images were obtained with an anti-GSDMD antibody (GSDMD). Nuclei were counterstained with Hoechst 33258 (Hoechst) after treatment with 20 μM GGA for 8 h in the presence or absence of 5 μM VIPER. Control cells were treated with the vehicle alone for 8 h. Merged images (merge) were constructed by tracing cell membranes on DIC images. The amplification bar represents 50 µm.

Next, we analysed the dynamics of GGA-induced cell death by measuring LDH activity in the culture medium. As a result, LDH activity was increased gradually and slightly in the medium until 5 h after addition of GGA, but rapidly increased at 8 h and reached the maximum at 24 h (Figure 4A). The time-dependent change in LDH activity was completely different from that of CASP4 activity after GGA treatment (Figure 3B). Hence, we measured the enzymatic activity of CASP1, another caspase for a lytic form of programmed cell death. As shown in Figure 4B, CASP1 activity was increased immediately and gradually after GGA treatment until 5 h, which was enhanced markedly at 8 h and remained at a high level until 24 h. The kinetics of the cellular CASP1 activity after GGA addition was surprisingly similar to those of LDH activity in the medium, suggesting that the cellular CASP1 activity was linked directly to GGA-induced cell death.

GGA-induced up-regulation of cellular CASP1 activity at 8 h was prevented significantly by cotreatment with oleic acid, VIPER, or NLRP3 inflammasome inhibitor MCC950 (Figure 4C), indicating that putative TLR4-mediated signalling (e.g., activation of inflammasomes) through UPR is indispensable for activation of pro-CASP1. Furthermore, notably, Figure 4D clearly shows that cotreatment with CASP4-specific inhibitor Z-LEVD-FMK blocked GGA-induced activation of CASP1 activity, suggesting that activation of CASP4 may be an upstream signal for activation of CASP1. It was also noteworthy that α-tocopherol prevented GGA-induced up-regulation of CASP1 activity (Figure 4C), although the vitamin did not block GGA-induced TLR4-mediated UPR (Supplementary Figure S3).

After 8 h of treatment, most of the plasma membrane and cytoplasmic space were occupied by GSDMD fluorescence (Figure 4E), which was also consistent with the biochemical finding of the N-terminal fragment of GSDMD at its maximum at 8 h (Figure 3D). The cotreatment with VIPER blocked GGA-induced plasma membrane translocation of GSDMD (Figure 4E). These results strongly indicate that GGA induces TLR4-mediated pyroptotic cell death in human hepatoma cells.

### GGA induces priming of the NLRP3 inflammasome

**Figure 5:**
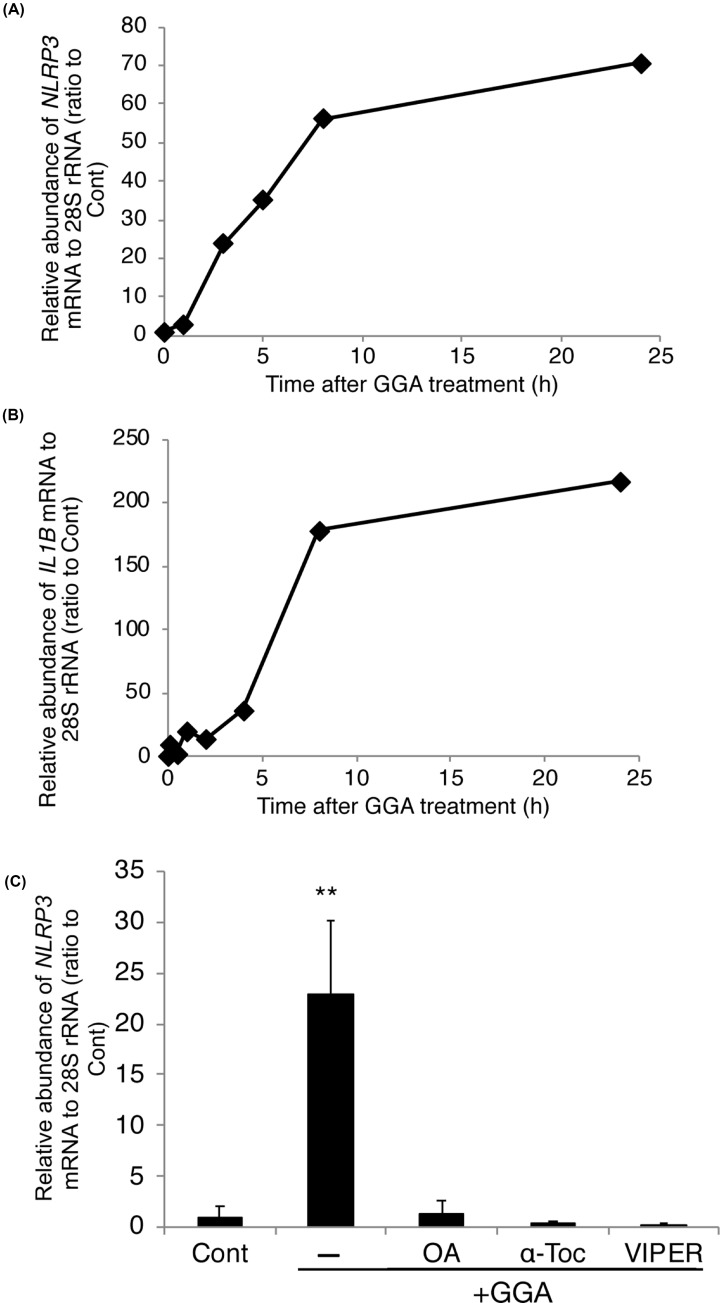
GGA induces priming of the NLRP3 inflammasome through TLR4 Total RNA was extracted at the indicated time points after 20 µM GGA treatment to estimate the levels of *NLRP3* (**A**) and *IL1B* (**B**) mRNAs by RT-qPCR with the internal reference control 28S rRNA. (**C**) The level of *NLRP3* mRNA was measured by RT-qPCR at 5 h after 20 µM GGA treatment in the absence (−) or presence of 50 µM oleic acid (OA+GGA), 100 µM α-tocopherol (α-Toc+GGA), or 5 µM VIPER (VIPER+GGA). Values are the means ± SD (*n*=3). ** indicates statistical significance (*P*<0.01) compared with controls as determined by the Student’s *t* test.

In addition to the aforementioned TLR4/UPR/CASP4 axis, it has been well established that the NLRP3 inflammasome is involved in TLR4-mediated cell death [20]. As a downstream component of TLR4 intracellular signalling, NLRP3 inflammasome priming requires transcriptional up-regulation of *NLRP3* gene expression. As shown in Figure 5A, GGA increased the mRNA level of the *NLRP3* gene dramatically by 70-fold at 24 h. The *IL1B* gene, another target gene of inflammasome priming, was more up-regulated by 220-fold (Figure 5B). The GGA-induced up-regulation of both genes was dependent on time, but the *NLRP3* gene response was slightly faster than the *IL1B* gene response.

The GGA-induced priming of the NLRP3 inflammasome was blocked by cotreatment with oleic acid (an authentic inhibitor of lipid-induced UPR), α-tocopherol (lipid-soluble antioxidant vitamin), or TLR4-specific inhibitor VIPER (Figure 5C). As described above, these three organic compounds all prevented GGA-induced cell death, suggesting that NLRP3 inflammasome priming may be strongly linked to GGA-induced cell death.

### GGA-induced mitochondrial hyperproduction of ROS coincides with NF-κB activation

**Figure 6:**
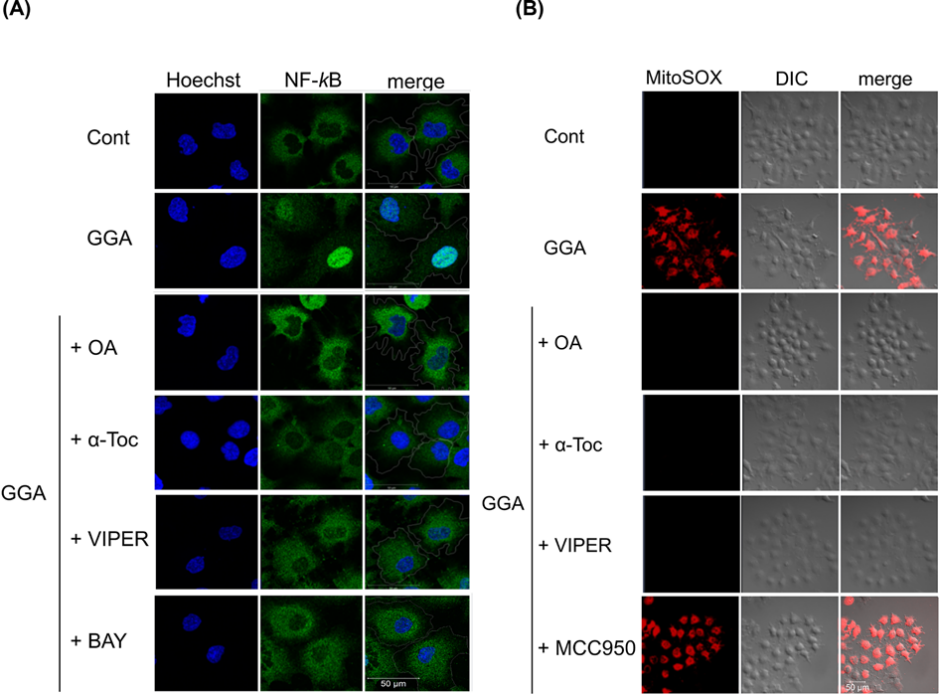
GGA-mediated hyperproduction of mitochondrial superoxides induces nuclear translocation of NF-κB through TLR4 signalling (**A**) HuH-7 cells were cultured under the following conditions: no treatment (Cont), 20 μM GGA alone (GGA), or 20 μM GGA with either 50 µM oleic acid (GGA+OA), 100 µM α-tocopherol (GGA+α-Toc), 5 µM VIPER (GGA+VIPER), or 1.25 µM BAY (GGA+BAY). Immunofluorescence images were obtained for NF-κB (green), and nuclei were counterstained with Hoechst 33258 (Hoechst) after 3 h of treatment. White lines in the merged images (merge) indicate the periphery of individual cells drawn with a respective DIC (differential interference contrast) image underlay. (**B**) Live cell imaging was performed with red fluorescence for MitoSOX under an LSM700 confocal laser-scanning fluorescence microscope at 15 min after each treatment described in (A), except that BAY was replaced with MCC950 (5 µM).

Because *NLRP3* gene expression is dependent on NF-κB [20], we examined subcellular localisation of NF-κB that usually remains with its inhibitor IκBα in the cytoplasm. However, upon activation, NF-κB moves to the nucleus and acts as a transcription factor. As shown in Figure 6A, NF-κB protein (p65) was present in the cytoplasm of control HuH-7 cells, whereas GGA clearly induced trafficking of the protein into the nuclear space at 3 h of treatment. The GGA-induced nuclear translocation of NF-κB was blocked by cotreatment with oleic acid, α-tocopherol, or VIPER. BAY 11-7082, an irreversible inhibitor of cytokine-inducible IκBα phosphorylation [36], also prevented the GGA-induced nuclear translocation of NF-κB, suggesting that GGA-induced TLR4 signalling is upstream of IκBα phosphorylation, but GGA-induced UPR alone may not trigger the phosphorylation. In 2013, BAY-11-072 was revealed to be a non-specific inhibitor of NF-κB activation, which acts by affecting ubiquitination [36]. Therefore, we inhibited NF-κB activation by BI605906, an IKK2 inhibitor of known specificity [37]. As a result, BI605906 also blocked GGA-induced nuclear translocation of NF-κB (Supplementary Figure S7).

Because we reported that GGA induces hyperproduction of superoxides in mitochondria at 15 min [6], we considered that GGA-induced hyperproduction of mitochondrial ROS might be important to induce nuclear translocation of NF-κB p65 protein and thus prime the NLRP3 inflammasome. When mitochondria-derived superoxides were observed using MitoSOX, all three compounds used in Figure 5C, which inhibited GGA-induced NLRP3 inflammasome priming, prevented mitochondrial red fluorescence accumulation in GGA-treated cells (Figure 6B), whereas MCC950, a selective inhibitor of NLRP3 activation [38], did not prevent GGA-induced hyperproduction of mitochondrial superoxides. These results suggest that a burst of mitochondrial superoxides or mitochondrial dysfunction is an upstream signal of GGA-induced NLRP3 inflammasome activation, which may be essential for nuclear translocation of NF-κB and thus priming of the NLRP3 inflammasome.

### Morphological alterations and intracellular calcium concentration changes after GGA treatment

**Figure 7:**
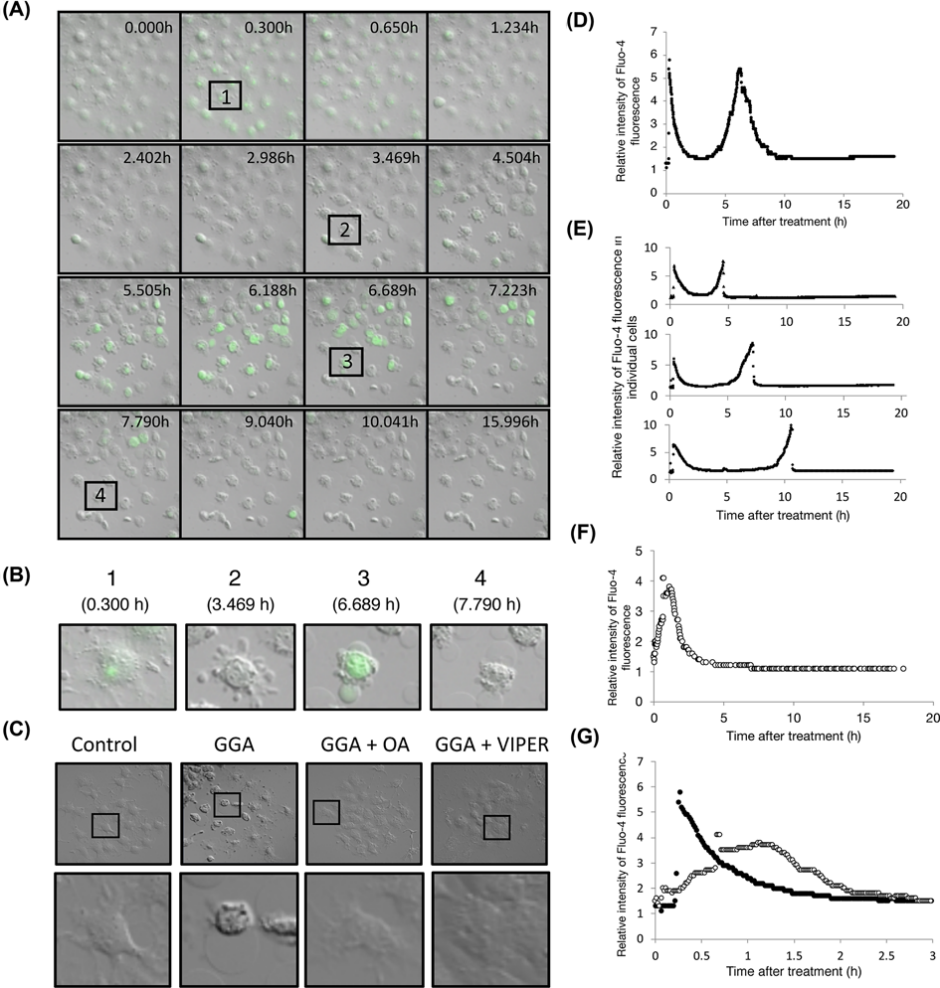
Time-series of live cell images of Fluo-4 AM-loaded HuH-7 cells after GGA treatment (**A,B**) Immediately after treatment with 10 µM GGA, live cell imaging was performed with the green fluorescence of Fluo-4 AM in HuH-7 cells cultured in a chamber unit of a LSM700 confocal laser-scanning fluorescence microscope. Snap shots at each time point shown in each frame were time-dependently arrayed from 0 to 16 h. Rectangles numbered 1–4 containing the same cell are enlarged and shown in (B). (**C**) Live cell imaging was performed after 16 h of treatment with ethanol (Cont), 10 µM GGA (GGA), 10 µM GGA plus 50 µM oleic acid (GGA+OA), or 10 µM GGA plus 5 µM VIPER (GGA+VIPER). Each square in each upper panel is enlarged and shown in the lower panel. (**D,E**) Total fluorescence intensity was monitored in each microscopic field (D) or each cell (E) and plotted up to 18 h every minute after GGA addition. (**F,G**) Total fluorescence intensity was monitored in each microscopic field and plotted up to 16 h every minute after GGA addition with Ca^2+^-free medium (F). Early changes in the fluorescence intensity of cells cultured in Ca^2+^-free medium (open circle) are shown from 0 to 3 h compared with HuH-7 cells (closed circle) cultured in DMEM containing Ca^2+^ (G).

Because GGA induced activation of CASP4/1 and fragmentation and plasma membrane translocation of GSDMD, resulting in TLR4-mediated pyroptotic cell death of HuH-7 cells, we next conducted the following experiment to obtain morphological evidence of pyroptosis. A time-series analysis was conducted using live cell images of Fluo-4 AM-preloaded cells from 0 to 16 h after GGA treatment (Figure 7 and Supplementary Movie S1). As shown in Figure 7A, a transient and immediate increase in the intracellular Ca^2+^ concentration was detected at 20 min after GGA treatment (panel 1 in Figure 7B). Next, the treated cells lost cell–cell adhesion and showed a reduced cell body size at 2–3 h after GGA addition (Supplementary Movie S1). A clear morphological change was detected at 3 h after adding GGA to the medium, which is shown in the 3.469 h panel of Figure 7A. Panel 2 of Figure 7B shows many irregular bulges protruding from the plasma membrane of a cell, some of which appeared to be separate from the cell, which were similar to apoptotic bodies. After 6 h, these irregular bulges fused together and remained attached to the cell membrane as two or three large balloon-shaped bulges (panel 3 in Figure 7B) with concomitant, secondary, and transient increases in the intracellular Ca^2+^ concentration. These balloons became larger than the original cell body size after 7 h (panel 4 in Figure 7B), which were maintained until 16 h. These morphological changes induced by GGA were prevented by cotreatment with oleic acid or VIPER (Figure 7C).

As mentioned above, the time-series analysis of the cellular Ca^2+^ concentration revealed two calcium concentration peaks after GGA addition to the medium. The first immediate and sharp peak was at 16 min and the second relatively broader peak occurred at approximately 6 h after the addition of GGA (Figure 7D). When we observed this in individual cells, the first peak appeared at 16 min in all cells, but the time at which the second peak appeared varied from cell to cell. The earliest was at 4.5 h and the latest was at 10.5 h (Figure 7E). Regarding the second peak, the average fluctuation of the fluorescence measured in the entire field of view over time was observed as a symmetrical peak at approximately 6 h after GGA treatment (Figure 7D). However, when individual cells were observed, the each second peak increased gradually, and when it reached the peak, it decreased rapidly, resulting in an asymmetric peak (Figure 7E).

When the cells were exposed to GGA in Ca^2+^-free DMEM, an early peak was detected within 1 h after GGA treatment, but no second peak appeared (Figure 7F). The first peak of the cytoplasmic Ca^2+^ concentration of cells cultured in Ca^2+^-free DMEM was delayed as a wider form compared with cells cultured in DMEM (Figure 7G).

## Discussion

In the present study, we investigated whether TLR4 signalling is involved as an upstream signal for GGA-induced UPR as well as a triggering signal for GGA-induced cell death. We demonstrated that GGA-induced UPR and GGA-induced cell death are both sensitive to cotreatment with VIPER, a specific inhibitor of TLR4 signalling, GGA-induced cell death is preventable by knockdown of *TLR4* gene expression, early transient activation of CASP4 is induced via GGA-induced UPR, and GGA stimulates NLRP3 inflammasome priming to activate CASP1 by NLRP3 inflammasome activation through TLR4 signalling. This induces pyroptotic cell death via translocation to the plasma membrane of the N-terminal fragment of GSDMD, a crucial target of active CASP1/4 during pyroptosis. The present results clearly highlight the significance of TLR4-mediated pyroptosis in GGA-induced cell death through activation of both CASP1 and CASP4.

### GGA activates the TLR4/UPR/autophagy (incomplete response) axis to induce pyroptotic cell death

GGA was reported initially as an acyclic retinoid that induces apoptosis in HuH-7 hepatoma cells [28], although caspase-3/7-specific inhibitor Ac-DEVD-CHO is unable to block GGA-induced cell death [2]. We found that GGA induced an incomplete autophagic response, suggesting that GGA induces autophagic cell death [6]. Although it was unclear how GGA killed hepatoma cells through autophagic events, we elucidated how GGA triggers a massive accumulation of initial/early autophagosomes. GGA induces an immediate ER stress response or UPR to form an upstream signal for induction of autophagy [7]. Thus, we speculated that GGA-induced UPR is linked to its lipotoxicity in human hepatoma cells.

In this context, it is important to understand how GGA induces UPR. To this end, we reviewed studies on UPR induction and noticed that TLR4 signalling triggers UPR induction in several different cellular systems [12,16,39–41] and TLR4 signalling is involved in inflammasome/CASP1-mediated cell death [42,43]. We previously reported that cotreatment with Ac-YVAD-CMK, a CASP1-specific inhibitor, blocks GGA-induced cell death in human hepatoma cells [2]. We therefore speculated that GGA stimulates TLR4 signalling, resulting in UPR and cell death. Indeed, as expected, we found that both UPR and cell death induced by GGA treatment were blocked by VIPER, a specific inhibitor of TLR4. VIPER is a virus-mimic 11-aa long peptide fused to a cell-penetrating delivery sequence, which is specific for TLR4 and inert in other TLR pathways [29]. VIPER prevents TLR4 signalling by interfering with interactions between TLR4 cytoplasmic domains and adaptor proteins, such as TIRAP (Toll/interleukin-1 receptor domain-containing adaptor protein/myeloid differentiation primary response gene 88 adaptor-like) and TRAM (Toll/interleukin-1 receptor domain-containing adaptor inducing interferon β-related adaptor molecule), by masking critical binding sites on adaptor proteins [29]. The TIRAP side of TLR4 signalling mainly leads to the production of proinflammatory cytokines [44], and the majority of anti-inflammatory cytokines signal via the TRAM side of the TLR4 receptor [45]. Therefore, we tentatively assigned the TIRAP side of TLR4 signalling or the myeloid differentiation primary response gene 88 (MyD88)-dependent pathway to inflammatory cell death [16]. In any event, Figures 1 and 2 clearly show that VIPER prevents both GGA-induced cell death and UPR, suggesting that GGA acts upstream from the cytoplasmic domains of TLR4. However, TAK-242, which also acts on the cytoplasmic domain of TLR4 [46], only partially inhibited the induction of cell death by GGA treatment, suggesting that the exact action points of VIPER and TAK-242 are different. Another TLR4 inhibitor, C34, inhibits TLR4 signalling by docking with the hydrophobic pocket of the TLR4 coreceptor myeloid differentiation protein-2 (MD-2) that is associated with the extracellular domains of TLR4 [47]. Cotreatment with C34 only partially suppressed cell death induced by GGA, suggesting that the action point of GGA might be close to that of MD-2.

GGA stimulates TLR4 signalling, therefore, we will next discuss how GGA stimulates TLR4 signalling. Several foreign and endogenous lipophilic materials, such as lipopolysaccharides (LPS) from Gram-negative bacteria [48], 7-ketocholesterol [16], oxidised low-density lipoprotein [49], and saturated fatty acids [50] including lauric acid [51], palmitic acid [52], and stearic acid [53], all act as TLR4 agonists/ligands and trigger inflammatory responses. However, the exact molecular mechanisms are still elusive other than under the assistance of LPS-binding protein and CD14, LPS stimulates the TLR4 receptor as an MD-2-bound heterodimeric complex in which two MD-2–LPS complexes bind two TLR4 extracellular domains resulting in ligation of two TLR4 molecules [54]. Analogous to the LPS mechanism, Nicholas et al. proposed that five molecules of palmitic acid associate with the hydrophobic binding pocket of MD-2 by hydrophobic protein modelling [52]. Because palmitic acid- and GGA-induced TLR4-mediated cell death are blocked by cotreatment with oleic acid, GGA may be a ligand of TLR4 adaptor MD-2 similar to palmitic acid. Another possibility is that GGA enhances the recruitment of TLR4 into lipid rafts of the plasma membrane. Indeed, Yamada et al. [55] and Cheng et al. [56] reported that TLR4 is recruited into lipid rafts upon palmitic acid treatment. Although not discussed in the literature, the palmitic acid-enhanced recruitment of TLR4 into lipid rafts may involve a fatty acid transport scavenger receptor protein, CD36, which is enriched in lipid rafts [57,58]. Because GGA targets lipid rafts during human immunodeficiency virus infection [59], it is reasonable to speculate that GGA might recruit TLR4 receptors into lipid rafts, which can be prevented by oleic acid treatment through CD36.

### TLR4 signalling-mediated mitochondrial ROS production is essential for GGA-induced cell death

In addition to the TLR4 antagonists used in the present study, α-tocopherol also efficiently inhibited GGA-induced cell death [2]. Of note in the present study, α-tocopherol also prevented GGA-induced intracellular events such as the hyperproduction of mitochondrial superoxides, nuclear translocation of NF-κB, and activation of CASP1 activity, most of which are also prevented by oleic acid or VIPER. However, among these compounds, the only antioxidative vitamin did not inhibit GGA-induced UPR, suggesting that GGA-induced UPR does not meet the necessary and sufficient conditions for GGA-induced cell death. The GGA-induced hyperproduction of mitochondrial superoxides was prevented by VIPER cotreatment, but was not suppressed by cotreatment with MCC950, indicating the signal for ROS production is located downstream of TLR4 and upstream of the NLRP3 inflammasome. West et al. reported that TLR4 signalling produces mitochondrial ROS via ubiquitination of evolutionarily conserved signalling intermediate in Toll pathways (ECSIT) protein after translocation of the TLR signalling adapter, tumour necrosis factor receptor-associated factor 6 (TRAF6) protein to mitochondria [60]. Recently, antioxidative protein peroxiredoxin-6 was reported to suppress TLR4-mediated mitochondrial ROS production by interrupting formation of the TRAF6–ECSIT complex induced by TLR4 stimulation [61]. Therefore, we speculate that GGA stimulates TLR4 to form TRAF6–ECSIT complex, which then results in the ubiquitination and degradation of ECSIT. A loss of ECSIT, a molecular chaperone for complex I of the respiratory chain, produces superoxides in mitochondria, which must be proved during GGA-induced cell death in the near future.

In this context, the inhibition of GGA-induced mitochondrial ROS hyperproduction by wortmannin, which we have previously observed in an autophagy study [6], can be interpreted relatively easily. Specifically, wortmannin is an inhibitor of Class III-PI3K, and we used this reagent as an inhibitor of autophagy by suppressing the action of beclin 1. As a result, the autophagy induction by GGA was inhibited, as expected, but the superoxide production by mitochondria was also immediately suppressed by wortmannin cotreatment with GGA [6]. Inhibition of GGA-induced autophagy by wortmannin could not explain why the increase in mitochondrial ROS production was suppressed [6]. However, if we speculate that wortmannin inhibited the downstream signal of TLR4 and prevented ECSIT ubiquitination, we can easily explain wortmannin-mediated suppression of GGA-induced mitochondrial ROS overproduction by our previous observation [6].

### GGA induces non-canonical and canonical pyroptotic cell death (Figure 8)

Pyroptosis is characterised morphologically by membrane blebbing and produces apoptotic body-like cell protrusions (termed as pyroptotic bodies) that fuse to a size as large as the cell body prior to plasma membrane rupture [62]. These morphological alterations were confirmed precisely by time-series recordings of live cell imaging (Figure 7 and Supplementary Movie S1). Membrane blebbing and pyroptotic bodies appeared at 3–4 h after GGA addition and the fused balloons were detected after 6 h. Of note, a transient increase in the cytoplasmic Ca^2+^ concentration was observed twice before and after these morphological changes of membrane blebbing/pyroptotic bodies and their fused balloons. Because the first Ca^2+^ peak was detected in GGA-treated cells that were also cultured in Ca^2+^-free medium, we believe that the first peak was caused by Ca^2+^ leakage from cellular Ca^2+^-storage organelles such as the ER. This is supported by our previous findings that GGA induced an ER stress response/UPR at 15 min [7]. The second peak was not detected in GGA-treated cells in Ca^2+^-free medium, indicating that cellular Ca^2+^ in the second peak must be from the medium. The role of each of the two increases in the Ca^2+^ concentration during GGA-induced pyroptosis is discussed individually later.

One of the most important findings of the present study is the GGA-induced early translocation of GSDMD to the plasma membrane (Figure 3D–F). Recent intensive studies on pyroptosis have revealed that the N-terminal fragment of GSDMD is the sole executor of pyroptotic cell death through its intrinsic pore-forming activity in the plasma membrane [24,27,28,63]. Immunofluorescence analysis in the present study revealed that most GSDMD signals were localised in the nuclear space and perinuclear region of control HuH-7 cells, which is consistent with the immunofluorescence data of GSDMD in several human cell lines in the Human Protein Atlas (http://www.proteinatlas.org/ENSG00000104518-GSDMD/cell). Next, the image analysis clearly demonstrated GGA-induced translocation of nuclear- and perinuclear-localised GSDMD to the cell membrane as early as 3 h after GGA addition. Consistently, we found the N-terminal fragment by Western blotting as early as 1 h after GGA addition (Figure 3D), when CASP1 was not yet activated (Figure 4B). GSDMD is an efficient substrate for both CASP1 and CASP4/5 [64]. In this respect, the activation of CASP4 observed at 1 h after GGA addition explains the unexpectedly early production of GSDMD-N, suggesting that GGA induced so-called non-canonical pyroptosis through induction of UPR, the resultant activation of CASP4, and then CASP4-mediated cleavage and plasma membrane translocation of GSDMD.

Interestingly, the translocated GSDMD signals were not distributed evenly throughout the plasma membrane. Specifically, the translocated GSDMD was particularly eminent in the concave parts of the plasma membrane or convex face of the inner plasma membrane (Figure 3F). This point becomes very interesting when considering that ESCRT-III assembly begins (nucleates) much faster on concave membranes during HIV budding [65]. Rühl et al. reported that Ca^2+^ influx through GSDMD pores serves as a signal for cells to initiate membrane repair by recruiting ESCRT machinery to damaged membrane areas [66]. The ESCRT-dependent membrane repair system may initiate upon the second increase in the cellular Ca^2+^ concentration during GGA treatment.

**Figure 8:**
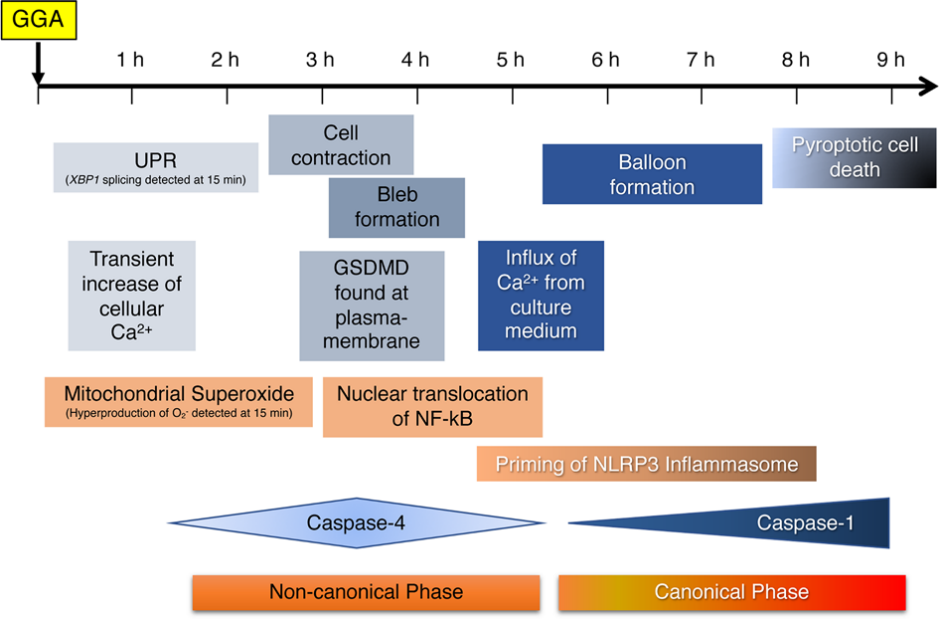
Timeline of the cellular events during GGA-induced cell death of human hepatoma HuH-7 cells GGA-induced cellular events described in the present study are arranged in a sequence of time when each event was detected in HuH-7 cells after addition of GGA to the culture medium, which does not necessarily mean to infer a causal relationship between events. Priming of the NLRP3 inflammasome means up-regulation of the mRNA levels of *NLRP3* and *IL1B* genes.

Next, we address how GGA treatment activates CASP4 in HuH-7 cells. In immunocompetent cells such as macrophages, CASP4 is activated directly by LPS in the cytoplasm. CASP4 binds directly to LPS and undergoes oligomerisation, resulting in activation of enzymatic activity [67], termed as non-canonical inflammasome activation [68]. Although we did not examine direct binding of GGA to CASP4 or its resultant oligomerisation, we presented experimental evidence that GGA-induced UPR activated CASP4 by demonstrating that thapsigargin, an authentic inducer of UPR, induced an active form of CASP4 by itself (Figure 3C). Thapsigargin is a non-competitive inhibitor of ER Ca^2+^-ATPase and induces UPR by causing Ca^2+^ release from the ER. Thapsigargin-induced UPR was not inhibited by cotreatments with oleic acid or VIPER (Supplementary Figure S6), indicating that thapsigargin-induced UPR is not linked to TLR4 signalling. Since Hitomi et al. [69] first reported that UPR activates CASP4 activity, studies have confirmed the UPR-mediated activation of CASP4 [70,71] and suggested a possible molecular mechanism involving CASP4 cleavage by Ca^2+^-activated calpain [72,73]. However, tunicamycin, another UPR inducer, inhibits N-glycosylation of nascent proteins in the ER and induces UPR, but does not release calcium, and did not activate CASP4 (Figure 3C). Therefore, GGA might activate CASP4 by putative calpain enzymes activated by the first Ca^2+^ peak at 1 h. Of note, GGA-induced activation of CASP1 was blocked by cotreatment with CASP4-specific inhibitor Z-LEVD-FMK (Figure 4C). Considering that Z-LEVD-FMK is unable to inhibit CASP1, we reasonably speculate that non-canonical activation of CASP4 via GGA-induced UPR is essential for canonical activation of CASP1. Therefore, non-canonical activation of CASP4 is an upstream signal of canonical activation of CASP1. As described above, we also speculate that active CASP4 may induce Ca^2+^ influx through GSDMD pores on the plasma membrane and further consider that the resultant intracellular Ca^2+^ surge (the second peak in Figure 7D, E) may be able to trigger NLRP3 inflammasome activation to produce active CASP1 (Figure 8). The resultant active CASP1 cleaves other target proteins including pro-IL-1β in addition to GSDMD and induces lytic cell death.

## Conclusion

In the present study, we report for the first time that micromolar GGA causes inflammatory cell death, pyroptosis, in human hepatoma HuH-7 cells via TLR4 signalling, where both mitochondrial hyperproduction of superoxides and nuclear translocation of NF-κB play essential roles to prime the NLRP3 inflammasome. We were also able to demonstrate that UPR-mediated non-canonical activation of CASP4 is essential to activate the NLRP3 inflammasome that produces active CASP1 during GGA-induced pyroptosis. Although the present findings are limited to a single cell line of human hepatoma-derived HuH-7, TLR4 was reported to have a pathological role during chronic hepatic inflammation and may be a cause of human hepatoma [74]. Hence, it is absolutely essential to prove GGA-induced pyroptosis in other human hepatoma cell lines to establish an anti-tumour effect of GGA against human hepatoma. In addition, a recent phase II clinical study reported prevention of hepatoma recurrence with peretinoin or 4,5-didehydro-GGA [75], suggesting further exploration of the preventive roles of GGA in hepatocarcinogenesis.

## Supplementary Material

Supplementary Figures S1-S7 and Supplementary Table S1

Supplementary Movie S1

## Abbreviations

Ac-DEVD-CHO: N-acetyl-aspartyl-glutamyl-valyl-aspartyl-aldehyde
Ac-YVAD-CMK: N-acetyl-tyrosyl-valyl-alanyl-aspartyl-chloromethylketone
CASP: caspase
DMEM: Dulbecco’s modified Eagle’s medium
ECSIT: evolutionarily conserved signalling intermediate in Toll pathways
ER: endoplasmic reticulum
GGA: geranylgeranoic acid
GSDMD: gasdermin D
HRP: horseradish peroxidase
IL1B: interleukin-1β
LDH: lactate dehydrogenase
LPS: lipopolysaccharide
MD-2: myeloid differentiation protein-2
NF-κB: nuclear factor-κB
NLRP3: NOD-like receptor family pyrin domain containing 3
ROI: region of interest
ROS: reactive oxygen species
TIRAP: Toll/interleukin-1 receptor domain-containing adaptor protein
TLR: Toll-like receptor
TRAF6: tumour necrosis factor receptor-associated factor 6
TRAM: Toll/interleukin-1 receptor domain-containing adaptor inducing interferon β-related adaptor molecule
UPR: unfolded protein response
