# Supplementary material for: TLR4-mediated pyroptosis in human hepatoma-derived HuH-7 cells induced by a branched-chain polyunsaturated fatty acid, geranylgeranoic acid
Source: Biosci Rep. 2020 Apr 28;40(4):BSR20194118. doi: 10.1042/BSR20194118 (PMC7189495; doi:10.1042/BSR20194118)
Supplement: Supplementary Figures S1-S7 and Supplementary Table S1 [file BSR-2019-4118_supp.pdf]

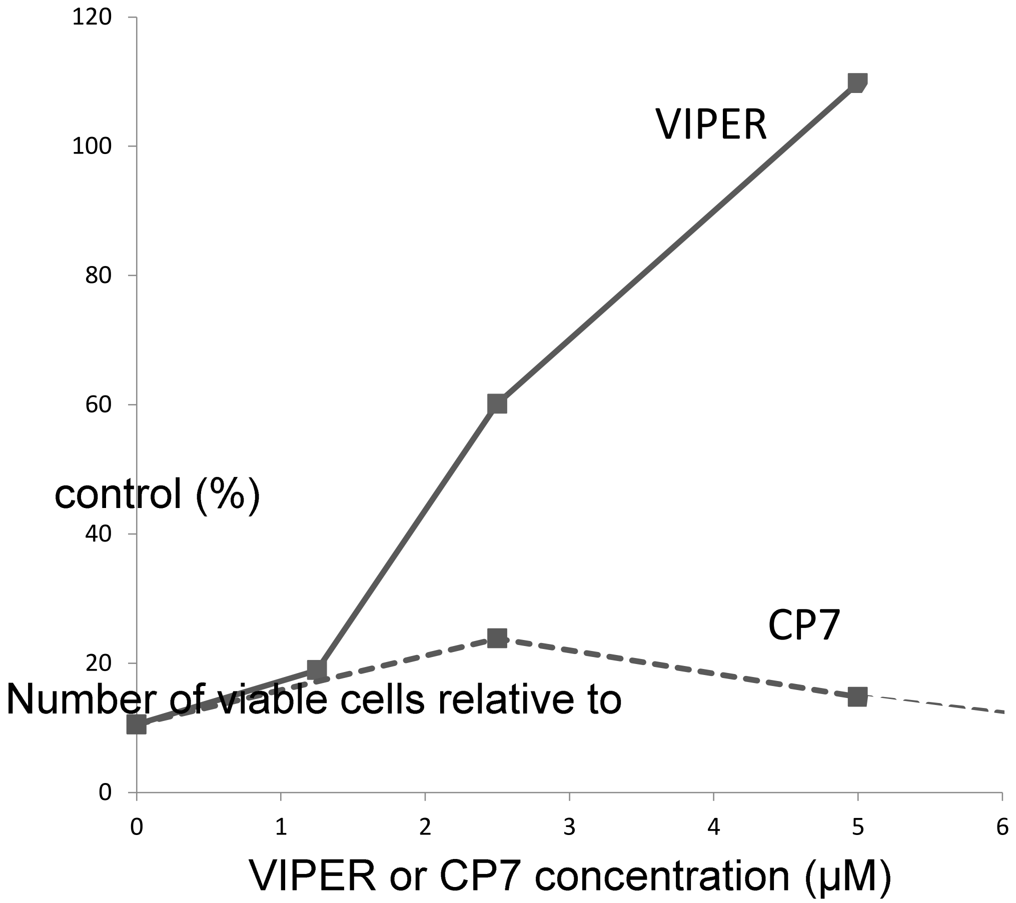


**Supplementary Figure S1. Dose-dependent inhibitory effect of VIPER on GGA-induced cell death.**

HuH-7 cells were treated with 20 µM GGA for 24 h in the presence of 0, 1.25, 2.5 or 5 µM VIPER (closed square and solid line) or CP7, control hepta-peptide (closed square and broken line).

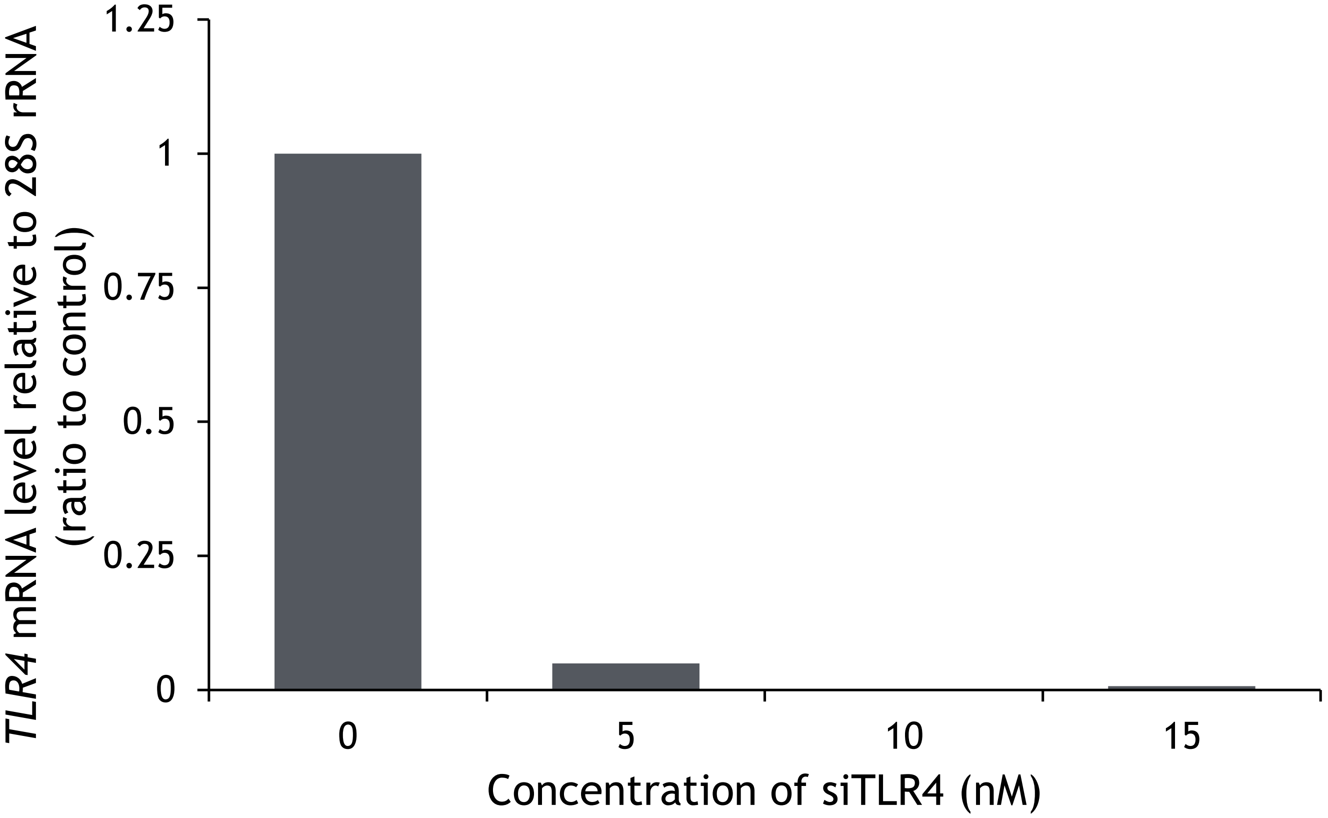


**Supplementary Figure S2. Knockdown of the *TLR4* gene expression with siTLR4.**

HuH-7 cells were treated with 5, 10, 15 nM *TLR4* siRNA (siTLR4) for 72 h. Total RNA was extracted to measure the cellular level of *TLR4* mRNAs by RT-qPCR.

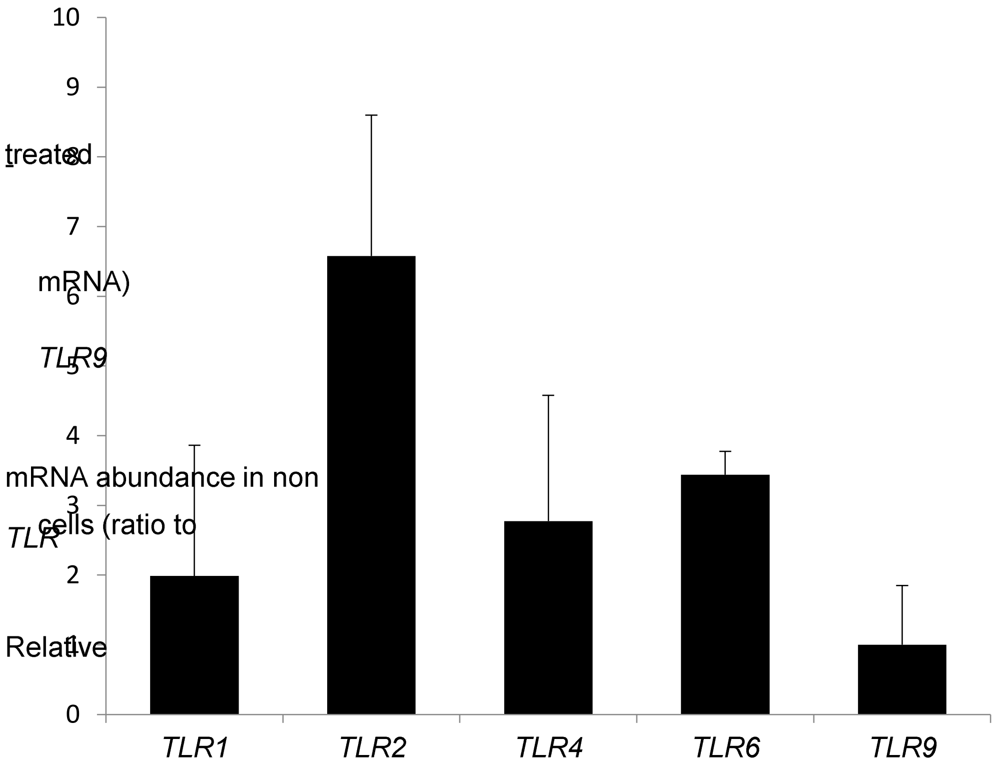


**Supplementary Figure S3. Relative TLR mRNAs abundance in control HuH-7 cells.**

Total RNA was extracted to measure the cellular levels of *TLR1*, *2*, *4*, *6* or *9* mRNA by RT-qPCR. The cellular baseline levels of *TLR* (*1*, *2*, *4*, *6* and *9*) mRNAs were plotted relative to the level of *TLR9* mRNA in control HuH-7 cells. Values are the means ± SD (n=3).

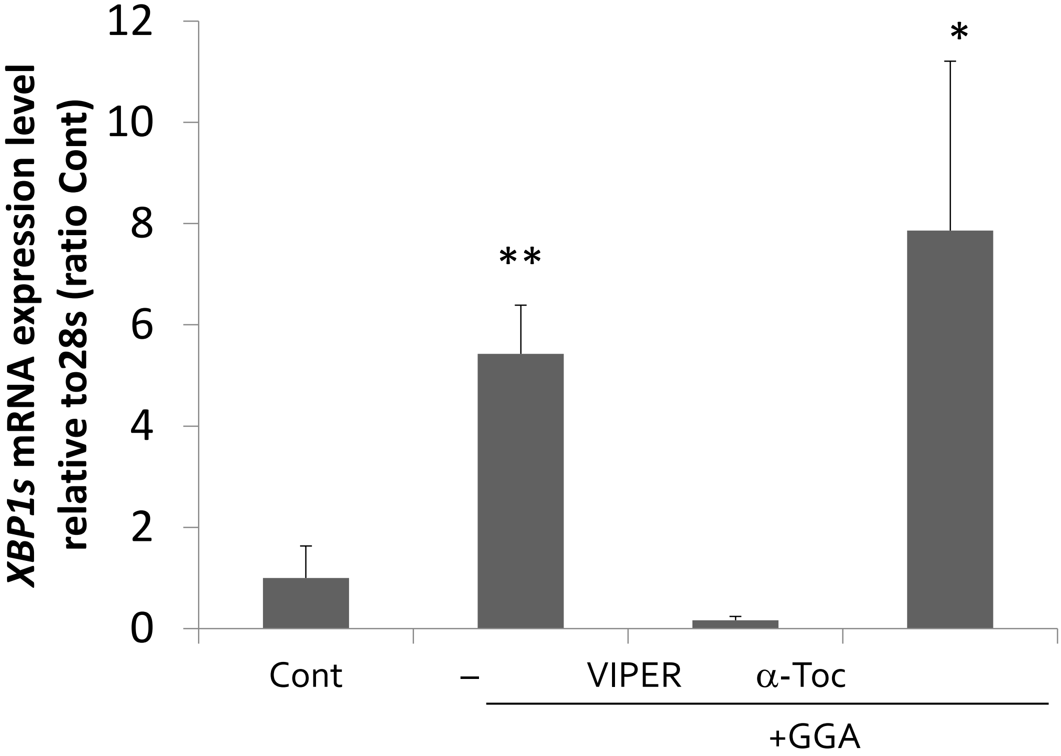


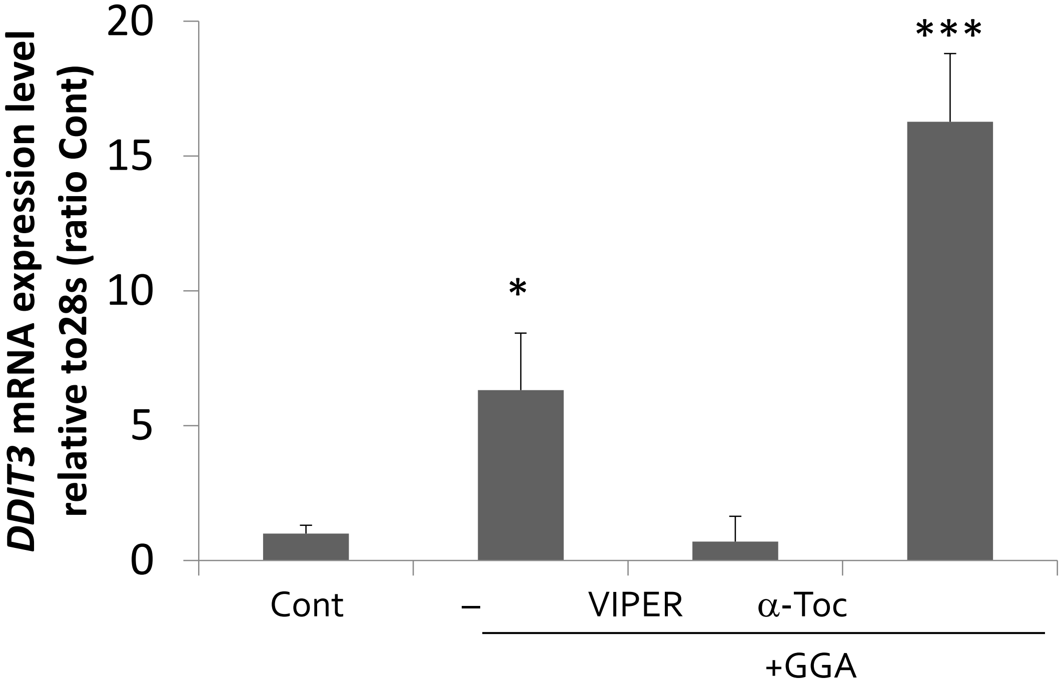


**Supplementary Figure S4. Effects of α-tocopherol or VIPER on GGA-induced UPR.**

HuH-7 cells were treated with 20 μM GGA for 3 h in the absence or presence of α-tocopherol or VIPER (100 or 5 μM, respectively). Total RNA was extracted to measure the cellular level of *XBP1s* or *DDIT3* mRNAs by RT-qPCR. *, **, *** indicate statistical significance ($p<0.05$, 0.01, 0.001, respectively), compared with each relevant control as determined by Student's *t*-test.

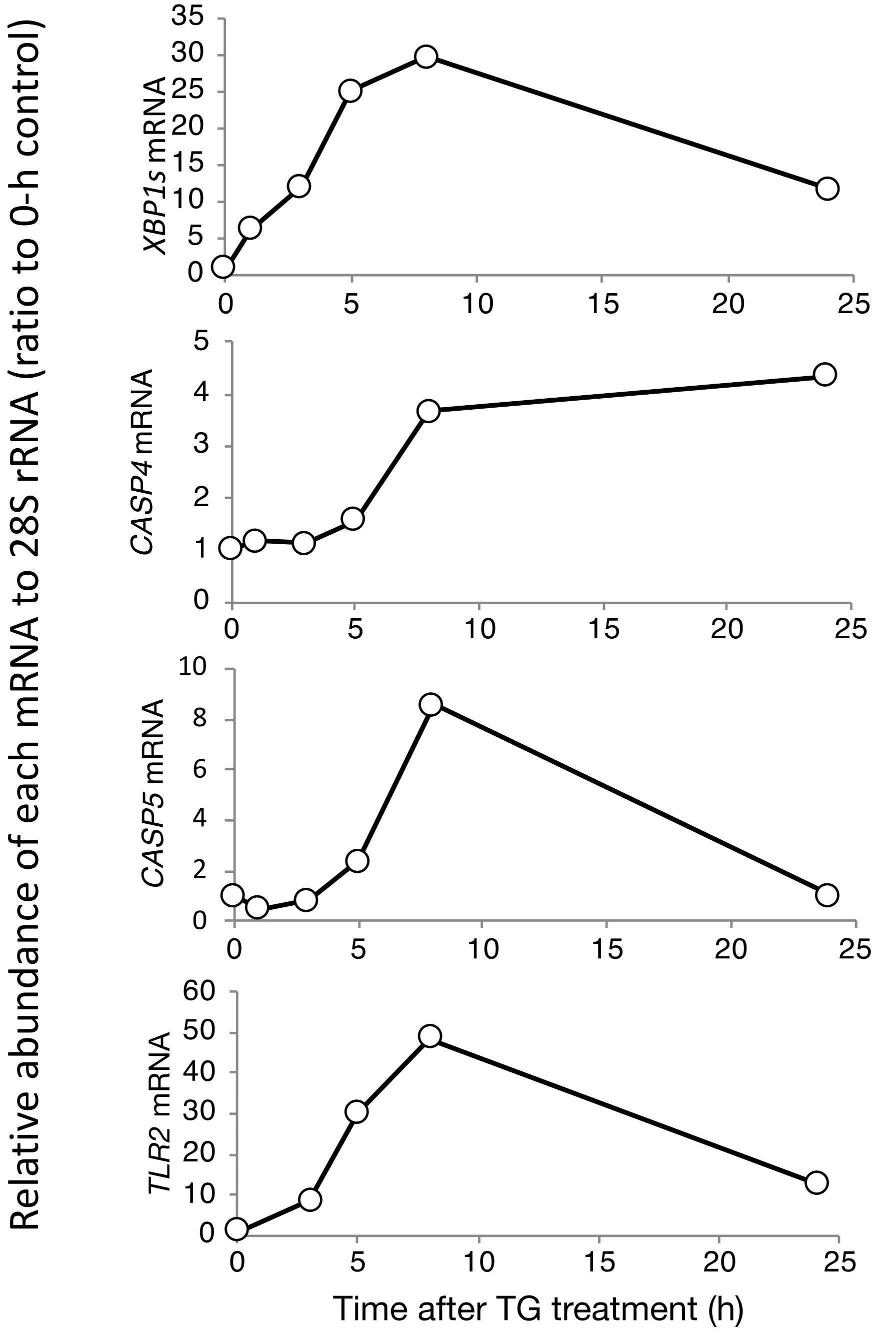


**Supplementary Figure S5. Thapsigargin (TG) induces upregulation of CASP4/5 and TLR2 mRNAs.**

HuH-7 cells were treated with 25 ng/mL TG, an inducer of UPR. Total RNA was extracted to measure the cellular level of *CASP4/5* or *TLR2* mRNAs by RT-qPCR.

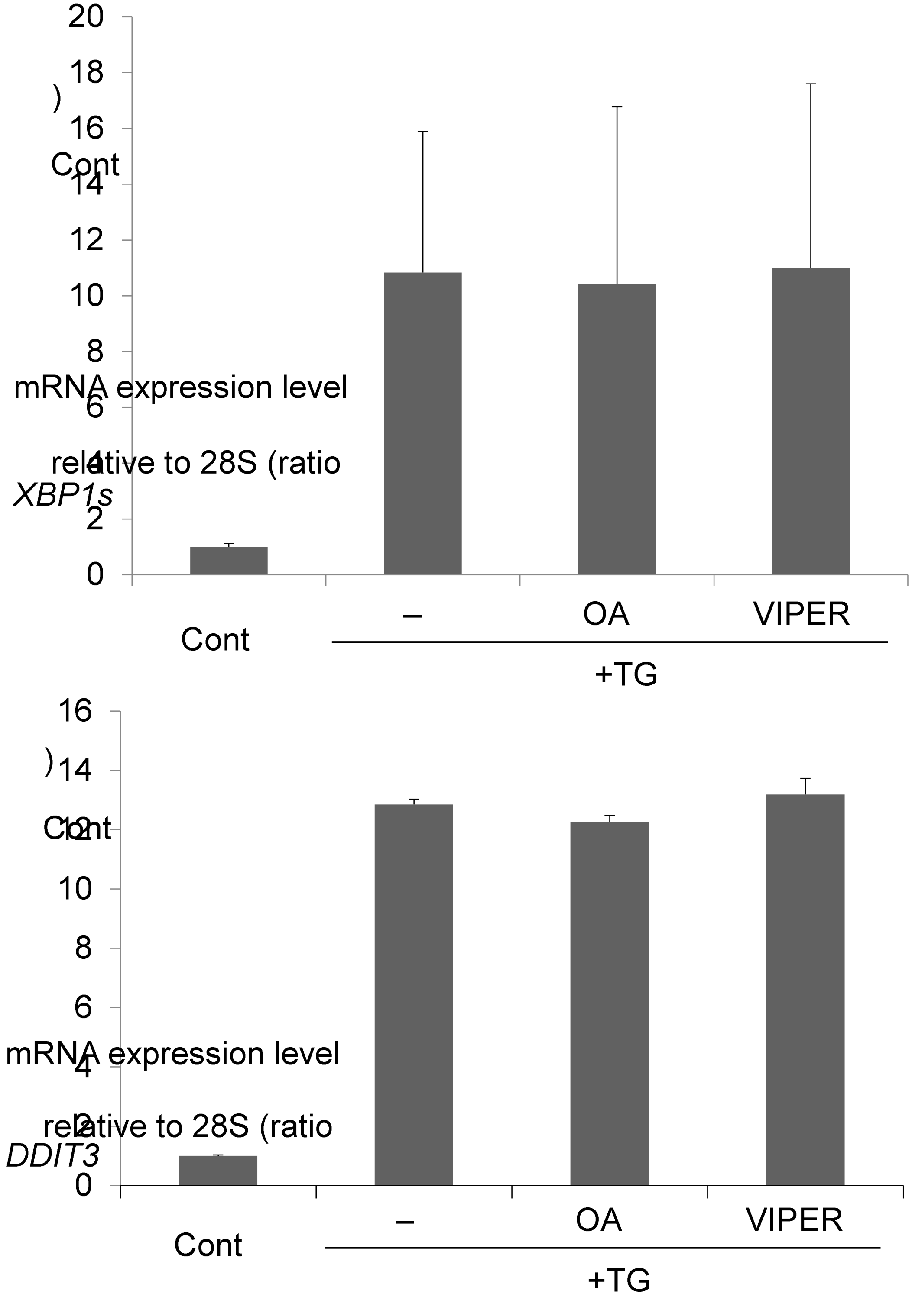


**Supplementary Figure S6. Effects of VIPER or oleic acid on thapsigargin-induced UPR.**

HuH-7 cells were treated with 25 ng/mL thapsigargin (TG) for 3 h in the absence or presence of OA or VIPER (50 or 5 μM, respectively). Total RNA was extracted to measure the cellular level of *XBP1s* or *DDIT3* mRNA relative to internal 28S rRNA by RT-qPCR.

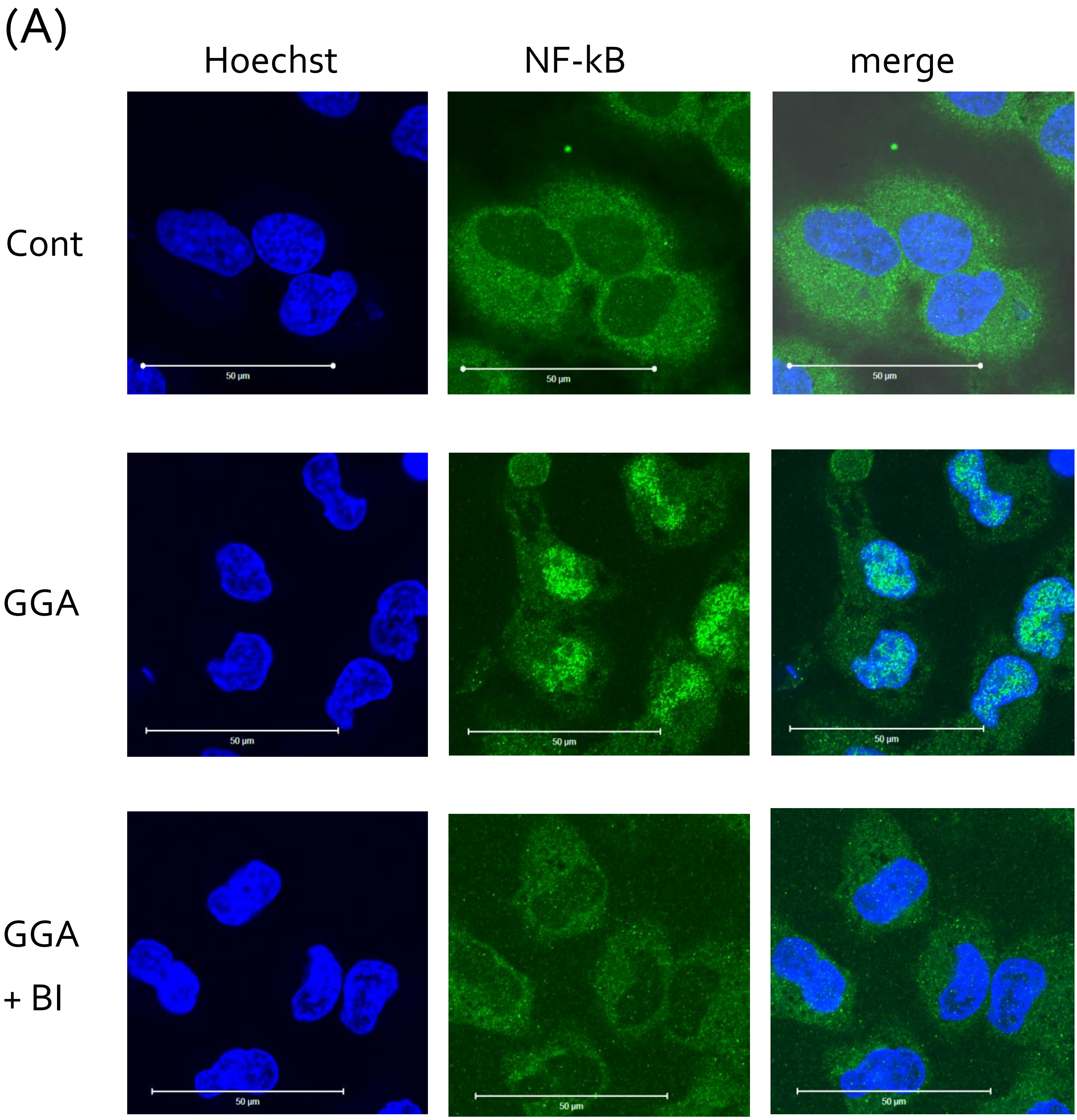


**Supplementary Figure S7. Effects of BI605906 on GGA-induced nuclear translocation of NF-kB protein.**

HuH-7 cells were treated with 20 μM GGA for 3 h in the absence or presence of 5 μM BI605906 (BI). Immunofluorescent images were obtained for NF-κB signals (green), and nuclei were counter-stained with Hoechst 33258 (Hoechst) after 3-h treatment. Objective lens x40 (A) and x20 (B).

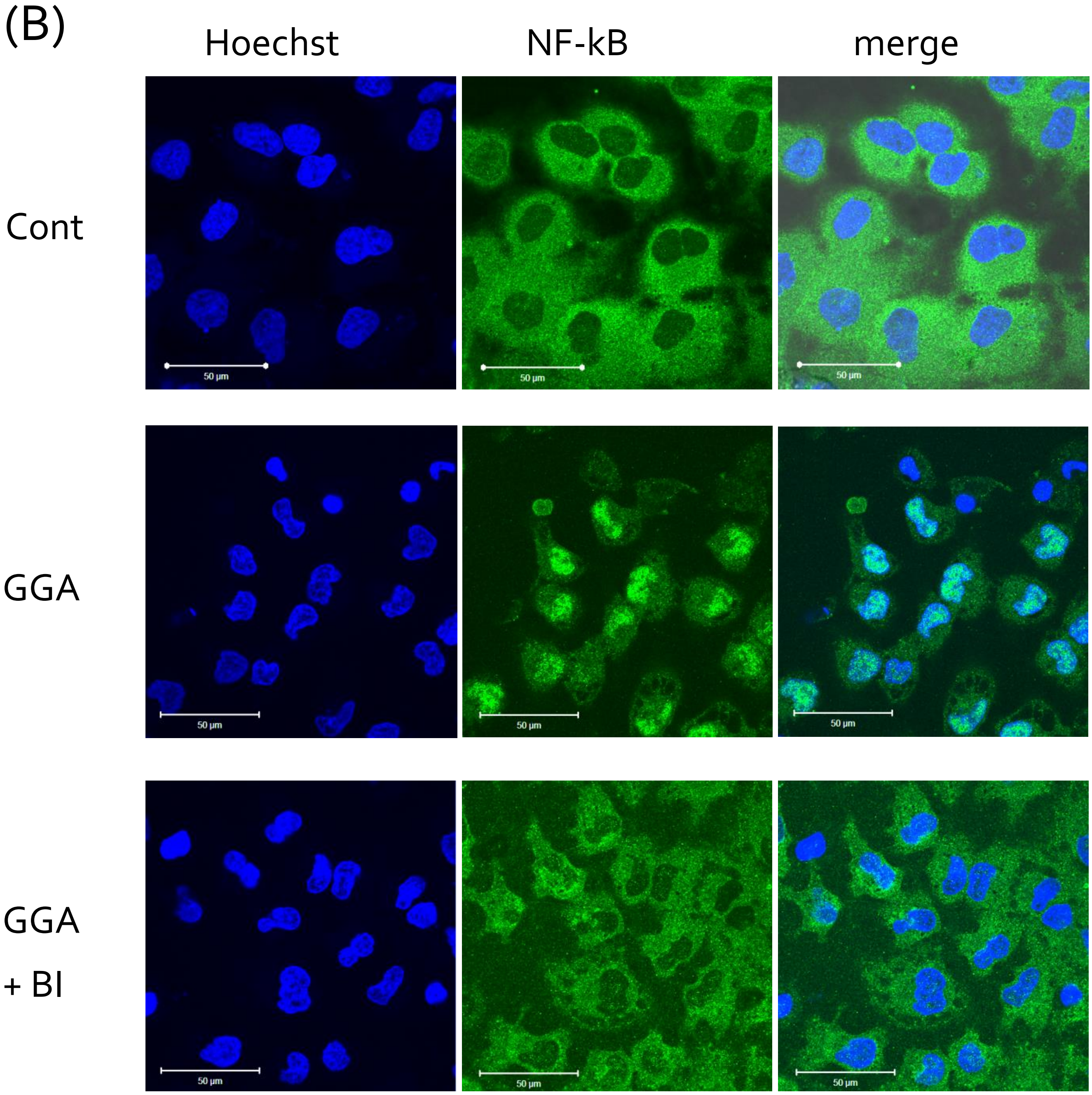


**Supplementary Figure S7. Effects of BI605906 on GGA-induced nuclear translocation of NF-kB protein.**

HuH-7 cells were treated with 20 μM GGA for 3 h in the absence or presence of 5 μM BI605906 (BI). Immunofluorescent images were obtained for NF-κB signals (green), and nuclei were counter-stained with Hoechst 33258 (Hoechst) after 3-h treatment. Objective lens x40 (A) and x20 (B).

## Supplementary Table S1. Primer List for RT-qPCR

| Genes | Primer | Sequence (5′-3′) |
|---|---|---|
| *CASP4* | F | TTGCTTTCTGCTCTTCAACG |
| | R | GTGTGATGAAGATAGAGCCCATT |
| *CASP5* | F | GTCTAAAGGACAAACCCAAGG |
| | R | TGTGAAGAGATGAGTGCCAAG |
| *NLRP3* | F | GTGTTTCGAATCCCACTGTG |
| | R | TGTGCTTCTCACGTACTTTCTG |
| *IL1B* | F | CCACAGACCTTCCAGGAGAA |
| | R | GTGATCGTACAGGTGCATCG |
| *XBP1s* | F | TGCTGAGTCCGCAGCAGGTG |
| | R | GCTGGCAGGCTCTGGGGAAG |
| *DDIT3* | F | ATGGCAGCTGAGTCATTGCCTTTC |
| | R | AGAAGCAGGGTCAAGAGTGGTGAA |
| *TLR1* | F | CCTAGCAGTTATCACAAGCTCAAA |
| | R | TCTTTTCCTTGGGCCATTC |
| *TLR2* | F | CGTTCTCTCAGGTGACTGCTC |
| | R | TCTCCTTTGGATCCTGCTTG |
| *TLR4* | F | CTGCCACATGTCAGGCCTTAT |
| | R | AATGCCCACCTGGAAGACTCT |
| *TLR6* | F | TGAAACAGTCTCTTTTGAGTAAATGC |
| | R | CAGAATCCATTTGGGAAAGC |
| *TLR9* | F | CCAGACCCTCTGGAGAAGC |
| | R | GTAGGAAGGCAGGCAAGGT |
| *28S rRNA* | F | TTAGTGACGCGCATGAATGG |
| | R | TGTGGTTTCGCTGGATAGTAGGT |
